# Thermosetting Polyurethane Resins as Low-Cost, Easily Scalable, and Effective Oxygen and Moisture Barriers for Perovskite Solar Cells

**DOI:** 10.1021/acsami.0c17652

**Published:** 2020-11-25

**Authors:** Matteo Bonomo, Babak Taheri, Luca Bonandini, Sergio Castro-Hermosa, Thomas M. Brown, Marco Zanetti, Alberto Menozzi, Claudia Barolo, Francesca Brunetti

**Affiliations:** † Department of Chemistry and NIS Interdepartmental Centre, University of Turin, Via Pietro Giuria 7, 10125 Turin, Italy; ‡ CHOSE (Centre for Hybrid and Organic Solar Energy), Department of Electronic Engineering, University of Rome Tor Vergata, Via del Politecnico 1, 00133 Rome, Italy; § S.E. Special Engines S.r.l., Strada del Cascinotto, 163, 10156 Torino, Italy; ∥ ICxT Interdepartmental Centre, Università degli Studi di Torino, Lungo Dora Siena 100, 10153 Torino, Italy

**Keywords:** perovskite solar cells, polyurethanes, encapsulation, oxygen barrier, water vapor barrier

## Abstract

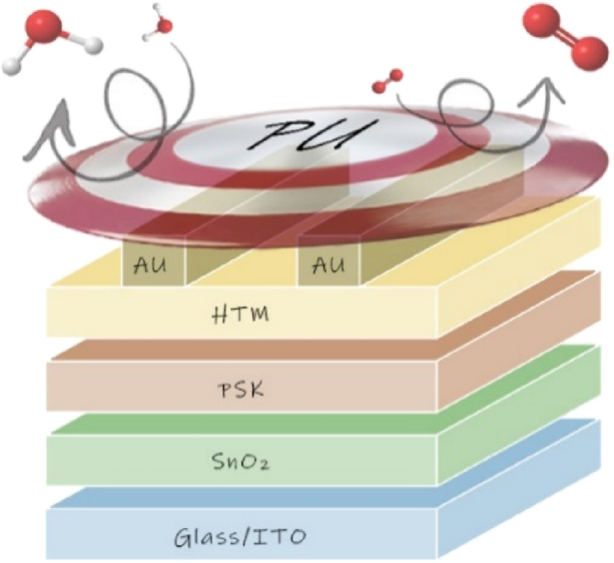

Long-term stability of perovskite solar cells (PSCs) is one of the main issues to be solved for forthcoming commercialization of this technology. In this work, thermosetting polyurethane (PU)-based resins are proposed as effective encapsulants for perovskite solar cells to prevent degradation caused by both moisture and oxygen. Application consists of drop-casting the precursor mixture directly over the devices followed by *in situ* polymerization, avoiding the use of other adhesives. PUs are cost-effective, lightweight, thermal, and light-stable materials whose mechanical, chemical, and physical properties can be easily tuned by thoughtful choice of their precursor. Encapsulated PSCs show extremely good stability when stored under ambient light (maximum, 1000 lux), controlled humidity (28–65%), and temperature (18–30 °C) by retaining 94% of the initial power conversion efficiency after 2500 h (4 months), whereas control devices lose 90% of their performance after 500 h (*T*_80_ = 37 h); once stored according to ISOS-D-1, PU-protected devices showed *T*_80_ > 1200 h. Encapsulated devices are stable even when immersed in pure water. The demonstration of PUs as promising solution-processed encapsulant materials for PSCs can pave the way for these to become a cost-effective encapsulation route alternative for future industrialization of this technology.

## 1 Introduction

In the past years, perovskite solar cells (PSCs) attracted huge attention from scientists involved in the photovoltaic field due to their impressive solar-to-electrical energy conversion efficiency.^1^ Indeed, PSCs remarkably outperformed other recent technologies (known as emerging photovoltaics), such as dye-sensitized solar cells (DSSCs)^2^ and organic photovoltaics (OPVs),^3^ and they have recently approached the efficiency of classical PVs, *e.g.*, Si-based devices. Additionally, PSCs can be effectively employed in different light conditions (from full sun to indoor illumination^4^) and in a plethora of different applications.^5^

Unfortunately, up to now, PSCs heavily suffer from instability due to intrinsic degradation^6^ of the materials used for the realization of the device and to extrinsic degradation related to the interaction with the environment.^7^ Indeed, both oxygen and moisture are external agents that can penetrate through the multilayer structure of the device to react with transport layers and perovskite crystals.^8^ These reactions, leading to lead iodide formation, cause the degradation of the perovskite layer and the loss of the photoelectrochemical properties of PSCs. Additionally, both perovskite and spiro-OMeTAD (*i.e.*, the most widely employed hole-transporting material (HTM) in n–i–p PSCs) partially suffer from extreme and prolonged UV irradiation.^9^ Domanski *et al.*(10) and Jena *et al.*(11) demonstrated that perovskite device performances deteriorate mainly because of the modification of the interface between the perovskite and spiro-OMeTAD at high temperature^12^ and of the gold migration through the hole-transporting layer (HTL), exposing PSCs to a temperature of 70 °C.^13,14^ In addition, the spiro-OMeTAD layer undergoes severe morphological deformation, showing large voids in it, which also reduces the cell performance.^15^ It is worth mentioning that, if protected in a black box with an inert gas-saturated atmosphere, PSCs show prolonged shelf-life stability.^16^ Yet, to extend their sphere of application from the laboratory scale to the industrial one, encapsulation of the device is mandatory.

Degradation rates have been shown to fall exponentially when effective encapsulation/permeation barriers can be developed and applied to perovskite solar cells.^17^ A good encapsulant/permeation barrier should provide the following features: (i) transparency over the entire solar spectrum to not limit the absorption of the device, in light of application in tandem and semitransparent stacks;^18^ (ii) wide thermal stability of the device experimented at temperature higher than 70 °C (*in operando*)^19^ and lower than −20 °C (during nighttime in deserts);^20^ (iii) good barrier properties toward both moisture and oxygen that cause the degradation of perovskite (PSK) and HTM layers;^21^ (iv) chemical inertness toward both contact and HTM (if applied onto the back of the cell);^22^ (v) resistance to UVA and UVB radiation (if applied on the front side) that usually caused the degradation of the PSK layer after prolonged exposure.^23^

As widely known, glass is one of the best encapsulating materials because, on the one hand, it possesses extraordinary barrier properties toward both oxygen and moisture (water vapor transmission rate (WVTR) and oxygen transmission rate (OTR) up to 10^–5^ g m^–2^ day^–1^ and 10^–4^ cm^3^ m^–2^ day^–1^ atm^–1^, respectively, when coupled with proper adhesives);^17,21^ on the other hand, it almost completely filters UV radiation.^24^ Yet, most glass covers need a sealant to completely encapsulate the device. This sealing process usually requires temperature higher than 80 °C and/or pressure application that could damage the devices. Additionally, most of the adhesives are not chemically inert toward the perovskite film.^17^ Moreover, when applied in PSCs, glass-based encapsulation almost doubles the overall weight of the device, leading to halving of the photoconversion efficiency/weight ratio.^25^ This could heavily jeopardize the feasible and cost-effective implementation at the industry scale. In this context, a great amount of effort has been recently made to realize glass-free encapsulation.^26^ For example, Dameron *et al.*(27) reported on the use of a multilayer of various metal oxides (*i.e.*, Al_2_O_3_ and SiO_2_) deposited by atomic layer deposition (ALD), one on the top of the other. Such an approach allows one to obtain very low WVTR and OTR (10^–5^ and 10^–3^, respectively), but the cost effectiveness of this deposition technique has still to be demonstrated. Recently, Choi and co-workers used ALD to deposit a 50 nm-thick layer of Al_2_O_3_ with good barrier properties (WVTR = 1.8 10^–2^ g m^–2^ day^–1^).^28,29^ Compared to inorganic encapsulant materials and related deposition processes, organic counterparts (*i.e.*, polymers and polymer-based composites) show some advantages such as tunable properties, lower production and deposition cost, and application on flexible devices.^30^ Nevertheless, they usually have relatively lower barrier properties, in most cases, not below 1 g m^–2^ day^–1^, if referred to a single polymeric material.^21^ Composite encapsulants, *i.e.*, organic–inorganic mixed films,^31^ could be also employed, but their synthesis is usually more expensive.^32^

Therefore, the development of a low-cost, effective, lightweight, and easily scalable encapsulant is a crucial, still unsolved, point for the forthcoming commercialization of PSCs.^33−36^ Scientists have mainly focused their attention on encapsulant materials with barrier properties reaching or exceeding 10^–5^ and 10^–3^ for WVTR and OTR, respectively. Yet, all materials that do not comply with these very strict requirements have been simply (and maybe too quickly) ruled out.^21^ It is worth mentioning that these values refer to preformed films to be sealed onto the device. However, for a liquid encapsulant directly casted on the device and then hardened, the effective barrier parameters can be substantially different from the self-standing film ones. It should be pointed out how the critical figure of merit is not the barrier property itself but the critical amount of water/oxygen reaching the device. Indeed, in ambient atmosphere, a WVTR of 10^–3^ g m^–2^ day^–1^ may cause the death of the device after 1000 days.^37^

With these considerations in mind, it is clear that there is not any report of the use of thermosetting polyurethane (PU)-based materials as encapsulants for photovoltaic devices, with their WVTR value being just acceptable (*i.e.*, 0.1–100 g m^–2^ day^–1^).^38^ However, albeit modest values of WVTR and OTR, this class of materials (aliphatic thermosetting PUs) possess some ideal features such as high transmittance (above 90%), chemical inertness, and good UV and thermal stability, if properly modified. Additionally, PUs are cost-effective, do not require cost-demanding polymerization procedure, and could be flexible and, therefore, very promising for flexible PSC and/or OPV and their mechanical and optical properties can be easily tuned by changing the precursors or by adding fillers/additives.^39^ Very interestingly, polyurethanes have been already applied as effective additives in the perovskite precursor mixture, leading to improved stability of the resulting layer.^40,41^ Polyurethanes are a peculiar class of polymers made by step-growth polyaddition (SGP) of isocyanates and alcohols to form a urethane bond. The mechanism of SGP consists of consecutive addition steps starting from the mixture of monomers (diisocyanates and bi- or multifunctional alcohols), leading to the formation of linear thermoplastic polyurethanes (TPUs) or branched thermosetting polyurethanes (PUs) of increasing molecular weight.^42^ In some cases, one of the precursors can be a multifunctional oligomer or polymer containing functional groups in its backbone. Last but not least, PUs can be easily recycled.^43^

Usually, thermoplastic polymers have been employed as co-encapsulants in conjunction with a glass slide or simply as edge-sealants. The most efficient polymeric encapsulants for PSCs and their performances are listed in Table S1 and compared with state-of-the-art encapsulant materials. According to Table S1, just a few reports referred to thermosetting polymers as stand-alone encapsulants. Among them, the best results have been obtained with UV-cured fluoropolymers.^44^ In all the other examples with thermoplastic polymers, the photovoltaic performances of the device are not even monitored. Using thermosetting PUs just as back encapsulants is likely arduous to reach the stability proposed by Bella *et al.* with F-polymers;^44^ notwithstanding, PUs possess key advantages of being cheaper, more easily recyclable than F-polymers, and more scalable.^43,45,46^ Indeed, thermosetting PUs have been actively used as protective polymers in electronic application (*e.g.*, LEDs).^47^ Moreover, they could be drop-casted or deposited by spin- or blade-coating onto the device in an open environment and they do not require any external source to induce the polymerization as it simply occurs at room temperature. As a matter of fact, the polymerization process could be sped up by the employment of relatively high temperature and energetic radiation that could, in turn, induce the degradation of the perovskite film. Very recently, Fu *et al*. already demonstrated the superior barrier properties of thermoplastic polyurethanes (TPUs) (compared to polyolefin elastomer (POE) and ethylene vinyl acetate polymer (EVA)) as co-encapsulant materials for PSCs.^48^ Indeed, they employed TPU as a sealing agent (*i.e.*, at the edge) in conjunction with a glass frit, which acts as the main encapsulant. It is worth mentioning that TPUs and PUs substantially differ in the polymerization geometry, with the former being linear and the latter being branched: therefore, TPUs are mainly employed as thermoplastic sealants, whereas thermosetting PUs should be polymerized directly in contact with the surface that has to be protected/encapsulated. However, the addition reaction occurs without the formation of any by-product molecules (*i.e.*, water, alcohols, and CO_2_): this is a key advantage of this class of polymers, opening the way to the application by drop-casting onto a photovoltaic device followed by *in situ* polymerization.

In this paper, we demonstrate, for the first time, how thermosetting resins based on polyurethanes (hereafter simply named polyurethanes) could be effectively employed as simple back encapsulants, providing an effective moisture and oxygen barrier, with the glass substrate acting as a front encapsulant. We specifically report on the application of PU as an encapsulant in planar direct PSCs. This type of architecture is particularly advantageous thanks to the low-temperature solution fabrication (<180 °C), which allows its application in flexible devices too.^49^

The encapsulation is performed, depositing two precursors onto the back of the device (*i.e.*, in contact with both HTM and Au) to ensure close interaction between the external layer of the device and the PU. We mainly focused our attention on moisture and oxygen protection more than on photochemical stability. Nevertheless, the encapsulated device showed also improved stability under continuous irradiation. Very interestingly, PU-encapsulated devices retain more than 94% of its initial efficiency after 2500 h (*i.e.*, >100 days) when stored in a laboratory environment. One should notice that the results reported throughout the present paper, albeit at the initial stage, allow one to classify thermosetting PUs as “worth-to-investigate” materials, contrary to what is usually stated by the perovskite community.

## 2 Results and Discussion

### 2.1 Selection of the PU

**Figure 1:**
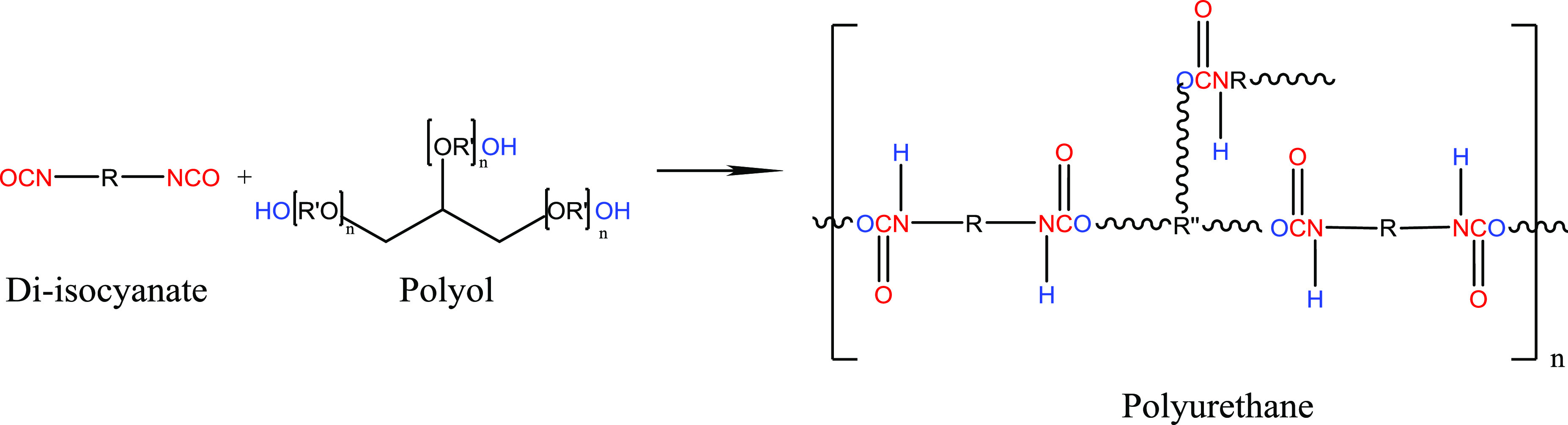
Schematic representation of the reaction to obtain polyurethane resins in which R and R′ have aliphatic structures and R″ is −[R′O]*_n_*–CH–CH[R′O]*_n_*–CH[R′O]*_n_*–.

Aiming at choosing the most suitable polyurethane-based thermosetting polymer, we screened six different bicomponent resins, obtained combining three different diisocyanate precursor (IC) mixtures mainly based on two aliphatic molecules (*i.e.*, hexamethylene-diisocyanate (HDI) and isophorone diisocyanate (IPDI)) and two polyol (PO) formulations (Figure 1). The PO and IC formulations differ in the prepolymerization degree and in the HDI/IPDI ratio, respectively, leading to polyurethane-based resins with tunable mechanical properties (see Section 4). The formation of the PU is a quite complex process: a relatively fast first step consists of a polyaddition reaction involving the majority of isocyanate moieties of IC and the hydroxyl groups of (partially prepolymerized) PO. This leads to the formation of a great amount of novel urethane bonds organized in long polymeric chains that are just poorly interconnected. The second step, characterized by a slower kinetic, consists of a limited number of addition reactions involving the already formed polymeric chains to obtain a three-dimensional and highly cross-linked polymer.^50^

The main properties of the six PUs obtained with various precursors are summarized in Table 1. The use of PO1 leads to less flexible (*i.e.*, higher Shore D) polymers compared to the one obtained with PO2. It is worth mentioning that the flexibility of the polymers could be further tuned by the thoughtful choice of the diisocyanate. Therefore, PO2 was selected as polyol precursors and polyurethanes obtained after the polymerization with the three ICs (namely, IC1, IC2, and IC3) were deeply investigated. Thermal stability of the polymers is just slightly influenced by the choice of the IC precursor: the decomposition temperature (*T*_d_) varies from 330.3 (PU21) to 327.7 (PU22) to 321.8 (PU23) according to the different HDI/IPDI ratios (see Figures S1–S3). Thermal degradation of all the polymers is completed at 400 °C. On the other hand, differential scanning calorimetry (DSC) evidenced meaningful differences in the glass transition temperature (*T*_g_) of different PUs: indeed, PU23 showed the lowest *T*_g_ (*i.e.*, 8.5 °C), whereas an increase was recorded for PU22 (*i.e.*, 13.3 °C) and PU21 (*i.e.*, 41.2 °C) as reported in Figures S4–S6. Straightforwardly, PU21 is discarded, having a *T*_g_ higher than room temperature, leading to a more complicated and less controllable deposition process. We also investigate the PU surface affinity for water. Albeit PUs are usually considered hydrophilic polymers, the hydrophilicity/hydrophobicity of thermosetting PUs strongly depends on both the reticulation degree of the polymers itself (*i.e.*, the number of unreacted alcoholic moieties) and the nature of the R and R′ groups in both the isocyanate and polyol precursors (straightforwardly, the ratio between polar urethane residues and apolar groups).^51^ Therefore, the simple definition of thermosetting PU as a hydrophilic material could be highly misleading. As a matter of fact, polyurethanes are commonly used as sealants and water proofers in various industrial applications.^46^ Moreover, the hydrophilicity/hydrophobicity is a property of the “surface” of the materials and it does not heavily impact its barrier properties. To check the hydrophobicity of our materials, we measured the contact angle (CA) of the selected film and a value higher than 100° has been found (Table 1). As thoroughly reported in the literature, a CA < 90° indicates a “mostly hydrophobic” material. More interestingly, once deposited on the film surface, the water drop is highly stable, and it does not show any tendency to flatten or to permeate the PU layer.

**Table 1:** Screening Parameters for the Thoughtful Choice of the Most Suitable PUs to Be Applied as Encapsulants in Perovskite Solar Cells

parameter	PU11	PU12	PU13	PU21	PU22	PU23
polyol formulation	1	1	1	2	2	2
diisocyanate precursor	1	2	3	1	2	3
viscosity at 25 °C (cP)	400	350	250	400	350	250
hardness at 25 °C (Shore D)	78	34	24	75	32	22
first polymerization time (h)	42	65	55	36	60	52
decomposition temperature (°C)				330.3	327.7	321.8
residue at 400 °C (%)				4.6	4.4	4.3
glass transition temperature (°C)				41.2	13.3	8.5
contact angle (°)				101.5	101.8	102.7

It is worth mentioning that the values reported in Table 1 are measured for self-standing films, whereas the final application consists of direct drop-casting of the precursor mixture onto the device followed by *in situ* polymerization. Therefore, some (slight) variations could be expected. Arising from the characterization summarized in Table 1, we identified PU23 to be the most promising polymer to be employed as an encapsulant in PSCs due to its lower *T*_g_, higher flexibility, and shorter first polymerization time (*t*_fp_) that can encourage an industrial and large-scale application. The *t*_fp_ is, in fact, the amount of time required for a roughly complete polymerization of the precursor mixture, allowing one to handle the sample and to easily delaminate it from the polymerization support.

### 2.2 Characterization of the Self-Standing PU Films

Before employing the selected polyurethane-based resin (*i.e.*, PU23) as an effective barrier in perovskite solar cells, we thoroughly investigated its optical and morphological features as well as its long-term stability under both thermal and UV light stress. All the stress tests were performed after the complete polymerization of the PU. The individualization of the actual time required for the complete polymerization is a crucial parameter to understand the aging curves of encapsulated PSCs (*vide infra*). Concerning PU23, a first polymerization step (making the film wieldy) occurs in roughly 2 days, whereas the polymerization process is completed after 1 week as proved by the differential scanning calorimetry (DSC) analyses (Figure S6) in which there is not any trace of postcuring phenomena.

**Figure 2:**
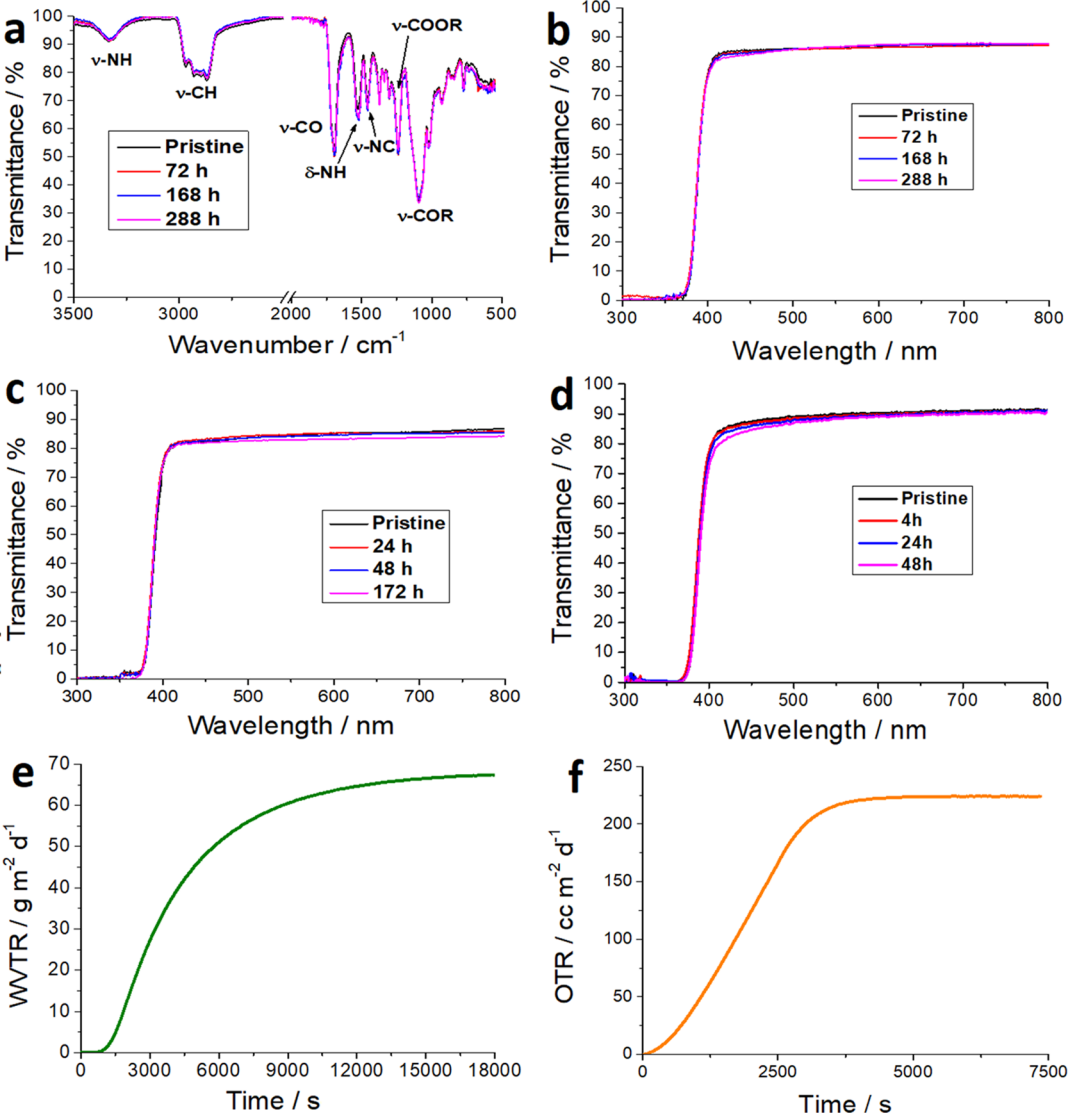
(a) ATR and (b) UV–Vis spectra of thermally (up to 100 °C) stressed films of PU23 for different times. UV–Vis spectra of (c) thermally (−50 °C) and (d) UV-stressed self-standing films of PU23. (e) Water vapor transmission rate (WVTR) and (f) oxygen transmission rate (OTR) of the self-standing film of polyurethane-based films (see Section 4 for further details).

We investigated the thermal stability of PU23 layer by treating it at relatively high or very low temperature (*i.e.*, in a conventional oven at 100 °C for different times or in a liquid N_2_ bath, respectively). We mainly focused our attention on the possible modification of (i) the optical response of the films (*i.e.*, transmittance of films in the UV–Vis region) and (ii) the chemical degradation of the polyurethane layer. The latter phenomenon could be quite easily monitored by means of ATR spectra in which the presence of NH bending signal of urethane group (located at 1460 cm^–1^) and the absence of the OH signal of the polyol precursors (located at around 3650 cm^–1^) are clear evidence of the retention of the polymeric matrix. As it is possible to see in Figure 2a, the thermal stresses, even prolonged up to a week, did not cause any modification in the structure of PU23. The peaks located at 3350 cm^–1^ and between 2850 and 3000 cm^–1^ are associated to the stretching of NH and CH bonds, respectively. Other meaningful peaks are the stretching modes of different moieties, *e.g.*, CO (1700 cm^–1^), NC (1460 cm^–1^), COOR (1240 cm^–1^), and COR due to R′ chain (1095 cm^–1^). The presence of NH is also confirmed by the band associated to its bending at 1530 cm^–1^. The three sharp bands located between 1300 and 1400 cm^–1^ are due to the presence of organic molecules (dissolved in the polyol formulation) behaving as UV stabilizers. Quite remarkably, the absence of the characteristic band of isocyanate group (at 2270 cm^–1^) in the spectra of pristine PU23 is a key proof to verify the complete polymerization of the film.

The degradation process of polyurethane matrix starts at about 200 °C, leading to partial volatilization of the polymer, due to pyrolysis and the formation of a charred residue thermally stable at 400 °C, as proved by thermogravimetric analyses (TGA, Figure S3). To verify if a prolonged thermal treatment (below 200 °C) could somehow induce irreversible degradation of the polymeric matrix, causing a substantial decrement in the barrier features of the PU, we recorded the UV–Vis spectra of a thermally aged sample: any possible change in the transmittance profile of PU23 could be ascribable both to damaging of the film or to degradation of the UV stabilizers. As it is possible to see in Figure 2b, the optical profile of PU23 remained constant even after 1 week (*i.e.*, 288 h) of aging at 100 °C.

The *T*_g_ of PU23 was evaluated by means of DSC and was found to be close to 9 °C. This temperature defines the cooling limit of 10 °C at which the PU film undergoes a hardening process and exhibits a decrease in its flexibility. This could cause some irreversible modification in the chemical structure of the polyurethane and/or some cracks throughout the film thickness. The occurrence of both cracks and matrix modification should be largely avoided to preserve the barrier features of the encapsulant. To verify the behavior of PU23 at very low temperature, we stored self-standing films of polyurethanes in a fridge (4 °C), freezer (−20 °C), and ultralow-temperature freezer (−80 °C) for 3 days. Interestingly, meaningful modification in both optical and morphological features could not be noted. Therefore, to further stress the PU23 film, we dipped it in a dewar (containing liquid nitrogen, −196 °C). The film integrity was checked by means of both optical microscopy and UV–Vis spectroscopy; if the film is damaged by temperature treatment, macrocracks would be easily highlighted by the microscope, whereas microcracks and/or modification in the polymeric matrix (*e.g.*, hypercross-linking of the polyurethane) would lead to a decrease in the film transmittance. After being removed from the dewar, the film was heated up at room temperature and then analyzed. Both optical microscopy and UV–Vis analyses (Figure 2c) did not show any substantial changes in the polymeric matrix. This demonstrates that, even if some changes occur during the low-temperature treatment, they are completely reversible.

Furthermore, we also investigated the possible self-standing film degradation caused by continuous UV irradiation. It is worth mentioning that, throughout this paper, we mainly focus our attention on the barrier properties (toward both moisture and oxygen, *vide infra*) of PU23. However, good resistance under UV stress is an added benefit to ensure the long-term stability of our encapsulant. We continuously stressed the self-standing film of the polymer under UV light for up to 48 h. This experimental setup is highly stressing because, during the test, the internal temperature of the chamber reaches 130 °C. Very interestingly, the sample did not show any clear modification in its optical features (Figure 2d). The value of the optical cut-off, *i.e.*, the wavelength at which the optical radiation is completely filtered, remains constant. As a matter of fact, any change in the latter may be considered a red flag of the degradation of UV protectors embedded in the polyurethane matrix.

Before using PU as an encapsulant material on the solar cells, we evaluated the barrier properties of our films toward both oxygen and water vapor. As clearly stated in Section 1, encapsulants should comply with very strict parameters. The experiments made on our samples yield a WVTR and OTR of 61.6 g m^–2^ day^–1^ and 256.6 cm^3^ m^–2^ day^–1^ atm^–1^, respectively (Figure 2e,f), measured with a MultiPerm instrument (see Section 4), showing inadequate values if compared to literature reports. Yet, our encapsulation approach is substantially different from the ones reported in the literature. In fact, the most common strategies employ barriers, which consist of self-standing films applied onto the device and sealed with specific glues (usually epoxydic ones), which have been shown to be very sensitive to handling and application procedures.^17^ Here instead, we directly drop-cast the precursor mixture on the device and let it harden. It should be pointed out that the barrier properties of the *in situ*-polymerized film could substantially differ from the self-standing films. Therefore, we attempted a more realistic evaluation, but an ITO-sustained film could not be analyzed with the same approach (see Section 4), with the glass being a better barrier. We also attempted a different method to estimate the WVTR, namely, the so-called calcium test in which the WVTR of a barrier is related to the (optical or electrical) degradation rate of a cutlet of calcium.^52^ Unfortunately, this mixture quickly reacts with calcium, making it impossible to determine any reliable value from these tests.

### 2.3 Application of PU Films onto the Multilayer Device

Once the good stability of PU23 under both thermal stress and UV irradiation was verified and knowing the intrinsic barrier properties (WVTR and OTR) of a 350 μm-thick self-standing film, we decided to test our thermosetting resin as an encapsulant in perovskite solar cells. Our approach consists of the deposition of the PU23 precursors liquid mixture directly onto the back of an n–i–p PSC, *i.e.*, over both the Au contact and HTM layer (in the space between the evaporated electrodes), and its polymerization *in situ* (see Section 4 and Figure S7). It should be pointed out that thermosetting PUs could be also easily spin- or blade-coated onto different substrates to more finely tune the thickness of the encapsulant layer. Nevertheless, to employ the latter approaches, further engineering of the small-area device is required; *i.e.*, the contact should be protected. Very interestingly, the HTM is substantially inert to the IC/PO mixture. This allows us to not protect the latter during the drop-casting of the encapsulant. The thickness of the completely polymerized PU23 layer was estimated to be 345 ± 23 μm.

Before starting the characterization of the complete devices, we investigated the effect of the direct polymerization of PU23 onto an incomplete stack (*i.e.*, TCO/SnO_2_/PSK/HTM). Notwithstanding the modest barrier properties measured, the effect of the addition of PU as an encapsulant material onto perovskite (PSK) is dramatically evident: after 2 days, the unencapsulated stacks suffer from severe yellowing of the perovskite layer ascribable to the degradation of the PSK crystals into lead iodide (PbI_2_). On the other hand, the encapsulated stack does not present any evidence of degradation even after 100 days (Figure S8). Encapsulated and unencapsulated stacks were characterized by means of X-ray diffraction (XRD, Mo K_α_ or Cu K_α_ source) to investigate the moisture-induced modification in the perovskite layer. The test was performed on a PSK layer deposited directly on glass/TCO substrates. Albeit the choice of two different X-ray sources could be somehow confusing to the reader, we were forced to do so. As a matter of fact, we employed (a more eneregetic) Mo K_α_ source to compare encapsulated and unencapsulated devices to minimize the shielding effect of the polyurethane film. Indeed, the latter is relatively thick (0.35 mm) if compared to the PSK film and, even if it is composed only by light nuclei (*i.e.*, H, C, O, and N), it partially masks the contribution arising from the active layer. Therefore, when we used (a less energetic) Cu K_α_ radiation to analyze the encapsulated film, both the (100) of the PSK and the (003) of PbI_2_ are completely masked by the broad peak ascribable to the (amorphous) polyurethane. On the other hand, we need Cu K_α_ when the aged films are investigated to enhance the surface sensitivity of the technique.

**Figure 3:**
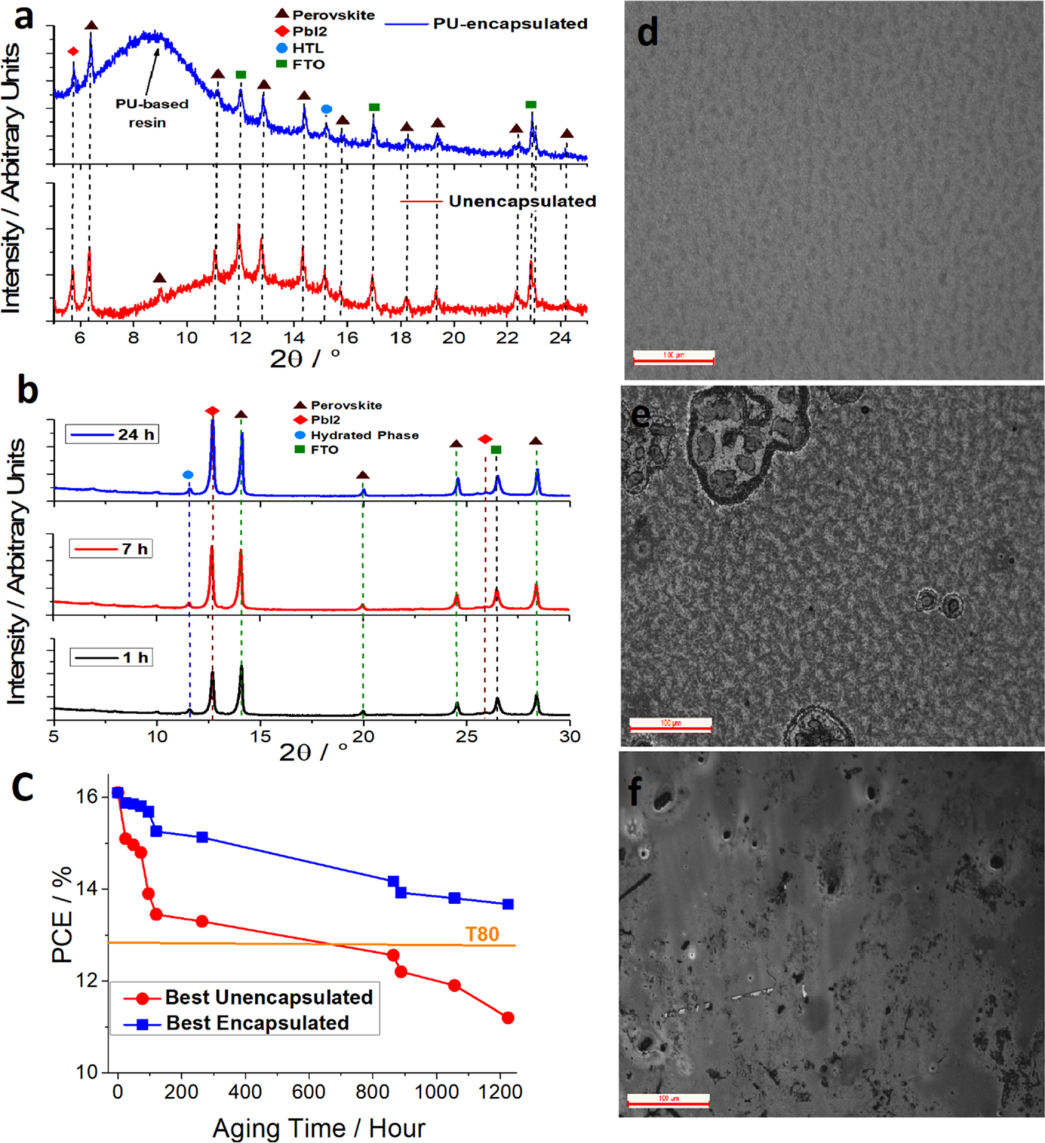
(a) XRD pattern of unencapsulated (red) and encapsulated (blue) devices after 2 days of storage in controlled atmosphere; Mo K_α_ was employed as an X-ray source to allow the investigation of a deeper section. (b) Diffractogram of unencapsulated samples after 1 h (black trace), 7 h (orange trace), and 24 h (purple trace) of aging time (stored at RH < 60% and *T* ranging from 20 to 30 °C); Cu K_α_ was employed as an X-ray source to perform a more surface-sensitive investigation. (c) Comparison between power conversion efficiency (measured under 1 Sun AM1.5G) *vs* aging time of unencapsulated (red dots) and PU23-encapsulated (blue squares) devices; aging was performed, complying with the ISOS-D1 standard. Optical microscopy images of unencapsulated PSK layer after (d) 1 h, (e) 50 h, and (f) 24 h of aging.

Before starting the measurements, the samples were stored under vacuum to minimize any interaction with air during the polymerization of PU23. After 2 days, the samples were removed from vacuum and analyzed. The diffractograms (Mo K_α_) of both stacks are reported in Figure 3a. Some characteristic peaks ascribable to the crystallographic planes of perovskite (ICDD card #00-003-1114) could be evidenced: (100), (110), (111), (200), (210), (211), (220), (221) and (300), (222), and (321) at 2θ = 6.38°, 8.99°, 11.05°, 12.80°, 14.32°, 15.74°, 18.23°, 19.30°, 22.36°, and 24.25°, respectively. Very remarkably, even though the unencapsulated sample was stored in vacuum during the polymerization of polyurethane matrix, it shows more marked degradation compared to the encapsulated stack as proved by the higher relative ratio between the peak centered at 5.76° and 6.38° (Mo K_α_) ascribable to the (003) plane of PbI_2_ and the tetragonal phase of perovskite (plane (100)), respectively. It is worth mentioning that Mo K_α_ allows one to investigate a deeper section of the samples, with more energetic radiation being compared to classical Cu K_α_ (0.717 and 1.540 Å, respectively). As a result of that, some peaks arising from TCO (planes (110) and (101) at 11.93° and 17.00°, respectively) and TiO_2_ ((101) plane at 15.19°) crystalline layers could be detected (green squares and light blue circles, respectively); the amorphous broadened peak between 2θ = 8° and 16° is due to the glass substrate. The latter is quite evident in the diffractogram of unencapsulated sample, whereas it is partially covered by the amorphous peak of polyurethane-based resin in the encapsulated sample.

The presence of the peak at 5.76° in both the unaged samples is due to incomplete conversion of PbI_2_ into a perovskite crystal. It should be pointed out that the presence of PbI_2_ peak in the pristine film does not necessarily point toward a degraded photoactive layer; indeed, it is, in small amounts, also beneficial for the PSC efficiency.^53,54^ Yet, the unprotected device shows a higher intensity of the latter peak if compared to the peak at 6.38° due to a quite fast degradation reaction occurring at the PSK/air interface. PbI_2_/PSK ratios of 0.32 and 0.65 are found for PU-encapsulated and unencapsulated stacks, respectively. This ratio remains almost constant for the encapsulated sample (Figure S9), whereas it dramatically increases in the exposed film (Figure 3b). To focalize the analysis on the modification of PbI_2_/PSK ratio, we employed less energetic radiation (*i.e.*, Cu K_α_), allowing one to avoid the scattering of the glass substrate and to magnify the contributions of the top layers (*i.e.*, lead iodide and/or perovskite).

In this context, we specifically investigated the modification of the relative intensity of the first crystallographic peak of both lead iodide and perovskite; the intensity of both peaks referred to the TCO (110) peak (26.48°, green squares in Figure 3b). The latter was employed, with a normalization reference being nominally identical in all the samples. The analyses were limited at 2θ = 30° angle (Cu K_α_). According to Bragg’s law, the change in the angles at which each reflection could be detected is due to the different sources employed for this second set of measurements. As evidenced from Figure 3b, unencapsulated samples present a diffractogram that is sensibly influenced by the moisture exposure time. Indeed, the intensity of some typical peaks of the tetragonal phase of perovskite layer (*i.e.*, (100) at 14.03° and (200) at 28.38°, brown triangles) gradually decreases, whereas the (003) peak due to PbI_2_ (at 2θ = 12.68°, red diamonds) becomes more and more meaningful. Very interestingly, the intensity of PSK’s (110) and (111) peaks also increases following the aging of the samples. This means that the hydrolyzation of the perovskite film leads to a preferential growth of the (100) and (111) planes to the detriment of (100) and (200) ones. Furthermore, some additional signals (11.57° and 25.53°) could be evidenced following the aging process. The attribution of the latter peaks is still being debated in the literature, but they likely are due to some hydrated perovskite phases.^55^ Other two small peaks, located at 2θ = 7.9° and 9.9°, could be ascribable to the 2D structure of the perovskite^56^ and they seem to be insensitive to aging (but their intensity is really low to make some quantitative analyses). Concerning the PbI_2_/PSK ratio (calculated from their more intense peaks), it changes from 0.33 (fresh sample) to 0.65 (sample being stored for 48 h under controlled atmosphere, red trace in Figure 3a) to 0.81, 1.07, and 1.52 after 1, 7, and 24 h of storage (at 25 °C and RH = 60%), respectively (black, orange, and purple traces in Figure 3b). The continuous aging of unencapsulated sample leads to the disappearance of PSK peaks after roughly 1 week, whereas the diffractogram of the encapsulated device remains unchanged over the same aging period (Figure S9). To further confirm the cause of the degradation of unencapsulated device, we performed some optical microscopy analyses after storing the sample under controlled atmosphere (*i.e.*, 25 °C and 60% RH). As recently proved by Di Girolamo *et al.* for CsPbBr_3_,^57^ the interaction of moisture within PSK crystals leads to an initial growth of the latter (during the first 48 h of exposure, Figure 3d) and to subsequent merging of the different crystallites (Figure 3e). This phenomenon becomes irreversible after roughly 100 h in a 60% RH environment (Figure 3f), which is characterized by the complete merging of the crystallites. The findings evidenced by optical analysis are also confirmed by the XRD spectra of aged samples (Figure 3b) in which the intensity of perovskite hydrated phase (peaks at 2θ =11.57° and 25.53°) increases with the exposure period. It is worth mentioning that the encapsulated device did not evidence any significant modification in either its morphological or crystallographic structure.

### 2.4 Performance of PU-Encapsulated Devices

Once the surprising barrier properties and inertness (toward both PSK layer and HTM) of the PU film were evaluated, we finally tested their effect on the PV performance of perovskite solar cells. The precursor mixture of the encapsulant was drop-casted onto the device in contact with both HTM and Au (see Figure S7) and left to polymerize for 1 day. Figure 3c shows the shelf-life measurements of the electrical parameters of different devices over a time span of 1200 h, stored according to ISOS-D1 (*i.e.*, room humidity and temperature). It should be pointed out that, to ensure an effective comparison between devices, the aging test was started when the polymerization of the barrier was almost completed. During that time, the devices are stored in a glovebox. Straightforwardly, *t* = 0 in Figure 3c corresponds to *t* = 1 day. The starting average efficiency of our cells (ITO/SnO_2_/perovskite/spiro-OMeTAD/Au) is 16%, which is in line with most of the articles in the literature for low-temperature planar n–i–p solution-processable PSCs.^58−60^ The unencapsulated devices show a more pronounced decrement in photoconversion performances compared to encapsulated counterparts, losing more than 10, 20, and 30% of their initial value after 100, 700 (this is the *T*_80_ value of unencapsulated device), and 1150 h, respectively. On the other hand, encapsulated devices do not reach *T*_80_ within the investigated time frame. Figure S10 shows the shelf-life measurements of the electrical parameters of different devices over a time span of 2500 h, stored in ambient conditions (*i.e.*, 28–65% RH and temperature ranging from 18 to 30 °C). In addition to this experiment, we performed another characterization on solar cells stored in the laboratory shield to evidence some eventual light-induced degradation phenomena of the PU film. Indeed, considering that thin-film perovskite solar devices have proven their potential to power indoor devices for applications such as smart homes, internet of things, etc.,^61^ that often do not need to last over 25 years but much less, stability improvement even in ambient conditions can be of significance. As expected, this aging test is more stressing than ISOS-D1. The unencapsulated devices show a decrement in photoconversion performances (Figure S10), losing more than 20, 50, and 80% of their initial value after 37 (this is the *T*_80_ value of unencapsulated device), 125, and 300 h, respectively. On the contrary, encapsulated devices retain more than 93% (best 96%) of their initial value over 2500 h. Concerning encapsulated devices, *T*_80_ was not reached within the investigated timespan, yet the so-called back-extrapolated *T*_80_ could give an effective estimation of this parameter:^62^ the latter is 6850 h (*i.e.*, 285 days), which is 185-fold the equivalent value of unencapsulated devices.

**Figure 4:**
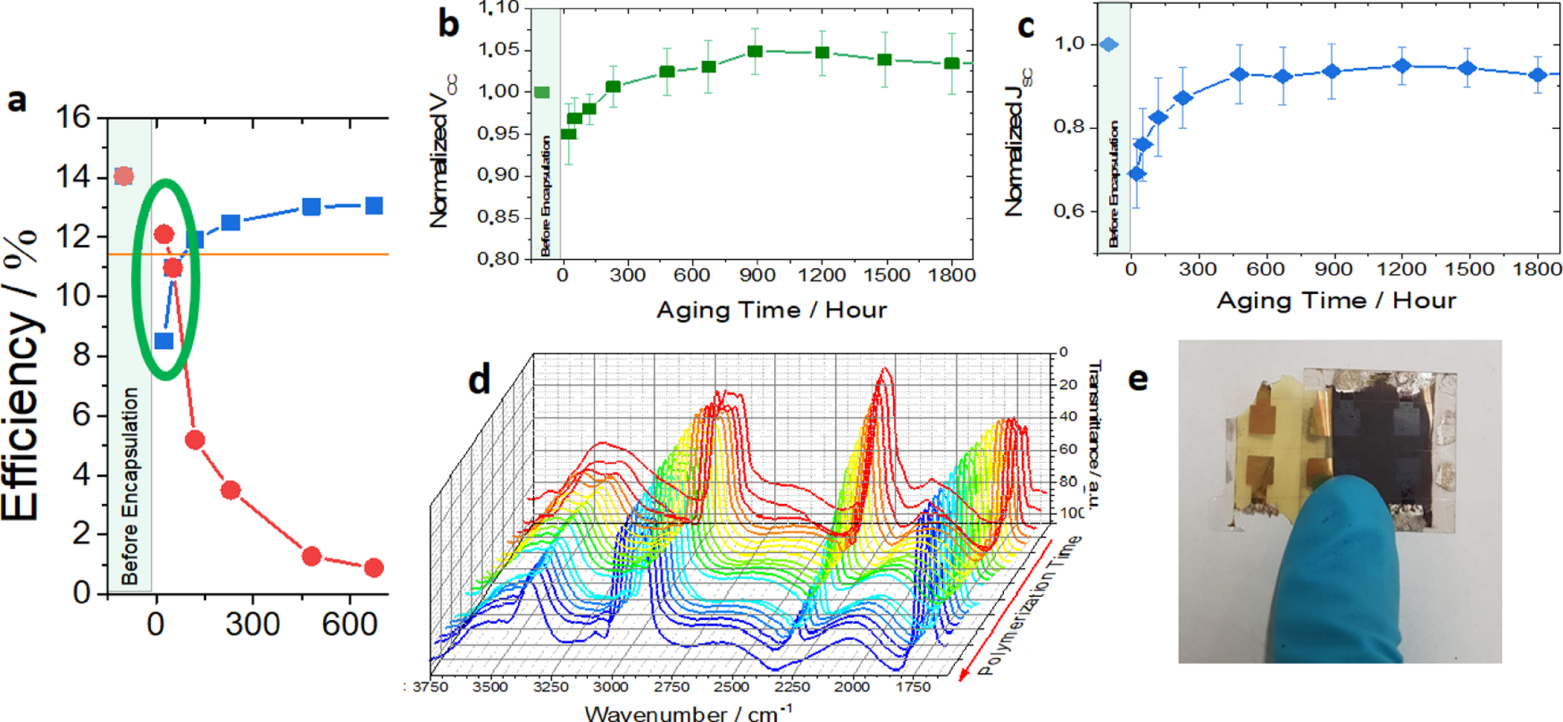
(a) Zoom of the first 600 h of aging for the most efficient encapsulated (blue squares) and unencapsulated devices (red dots); normalized photoelectrochemical parameters (*i.e.*, (b) *V*_OC_ and (c) *J*_SC_) of encapsulated devices within the aging period (measured under 1 Sun AM1.5G). (d) FTIR spectra of the polymerizing mixtures. (e) Digital photograph of the delaminated PU-based encapsulant.

Unencapsulated devices initially suffer a decrease in photocurrent (Figure S11a) followed by one in both FF and *V*_OC_ (Figure S11b,c), resulting in a drop of over 80% of their initial power conversion efficiency in just 300 h (Figure S11d). It is worth mentioning that the aging profile of PU23-encapsulated devices is not so straightforward: in the first days after the encapsulation, the photoconversion efficiency of the devices decreases faster than reference ones (showing a drop of 35%, Figure 4a). Then, after 2 days, there is partial recovery of the photoactivity and the electrical figures of merits became stable after 1 week, retaining their performances (93% of initial PCE) for 2500 h.

The initial drop of all the photovoltaic parameters is consistent with the time frame of the *in situ* polymerization process.^50^*J*_SC_ (Figure 4c) seems to be the most influenced among photovoltaic parameters by the polymerization process; compared to the fresh device, it experiments a severe decrease (−30%) probably due to the interplay between the liquid precursor mixture and the HTM (and the dopants in it) that may reduce the charge transport properties of the latter. This also impacts on the *V*_OC_ (Figure 4b) that is 5% lower than the fresh devices. Nevertheless, once the latter process ended (or at least substantially slowed down), *J*_SC_, *V*_OC_, and η rise again, approaching the values of pristine device (Figure 4b,c). Polyurethane polymerization is a complex thermoset process. Its kinetic is affected by factors like the composition of the reaction mass, type of catalyst, and possible side reactions.^63^ Remarkably, we found a strong correlation between the polymerization kinetic of polyurethane-based film (Figure 4d and Figure S12a) and the recovery trend of photovoltaic parameters. After the initial drop, as far as the rate of the polymerization process slows down (Figure S12b), the photoconversion efficiency of encapsulated device increases continuously. Once the polymer is completely cross-linked (Figure 4d), the photovoltaic parameters reach a constant value too. For further details on this correlation, see the Supporting Information (Figure S12 and related discussion).

This unprecedented behavior could be ascribed to an interaction between the encapsulant materials and the HTM. As a matter of fact, when deposited onto the device, the precursor mixture is still in its liquid form. It is reasonable to believe that the PU mixture cannot penetrate the gold electrode. However, it will get in contact with the uncovered portion of the HTM layer, which is easier to permeate for the PU in its liquid form. Once permeated into the HTM, it could diffuse throughout the layer, allowing strong adhesion of the material. Albeit further studies are obviously required to deeply understand the mechanism of device/encapsulant interaction, the HTM permeation hypothesis befits experimental results, as shown in Figure 4e: once mechanically delaminated from the device, the PU film appears to be yellow. The color is likely due to the HTM (*i.e.*, spiro-OMeTAD) strongly interacting with the polymeric encapsulant. A strong interaction between gold and PU is also expected, as already reported in the literature.^64^ This is particularly appealing considering the forthcoming application of PU-based resins in a large-area device in which the majority of exposed area is made by back contact. On the other hand, the PSK layer is still adherent to the TCO substrate. This proves that the encapsulation process does not directly influence the photoactive layer that is actually protected by the HTM (in the stack configuration) and both HTM and gold (in complete devices). Indeed, if the PU mixture would penetrate the HTM and interact with the perovskite layer, some morphological and phase modification of the latter could be fairly expected. Yet, as also proved by XRD analyses (Figure 3a), no changes could be evidenced following the encapsulation of the stack.

As it is possible to see from Figure 4b, after a stabilization period (first 250 h from the encapsulation), the open-circuit voltage of the encapsulated device is systematically higher than the value of the fresh device. In perovskite solar cells, the theoretical *V*_OC_ value is limited by the energy gap of the photoactive material (*i.e.*, the difference between conduction and valence bands of the perovskite film).^65^ Yet, the real value could be reduced by both dark current and recombination reactions.^66^ In our case, a modification in the perovskite electronic structure of encapsulated device is mostly unlikely, with the polymeric matrix being not able to penetrate the PSK layer (Figure 4e). Therefore, the higher *V*_OC_ is tentatively ascribed to a decrease in losses due to the interaction between polyurethane-based matrix and HTM layer and/or gold electrode. An enhancement in the quality of the PSK/HTM interface cannot be excluded. We are aware that, in the literature, a higher *V*_OC_ compared to the fresh device is also ascribed to different factors: Fei and Wang^67^ proved that the air exposure (48 h) of SnO_2_-based PSCs could lead to age-induced recrystallization of the perovskite layer, which suppresses both trapping and recombination of charges, ameliorating the transport properties of the active layer; Liu *et al.*(68) observed that controlled exposure (24 h) of spiro-OMeTAD to oxygen could lead to the formation of spiro-OMeTAD^+^ and to longer-living hole carriers; finally, Lee *et al.*(69) postulate bettering of the PSK/ETL interface with time due to the self-passivating properties of SnO_2_ reaching a plateau value after 100 h. Albeit these effects could not be completely ruled out, they are quite unlikely to be the reason behind the recovery of our devices, with the latter being stored in the dark for 3 days before encapsulating them. Therefore, if some of the former phenomena occurred, it will result in the PCE value of pre-encapsulation device.

**Figure 5:**
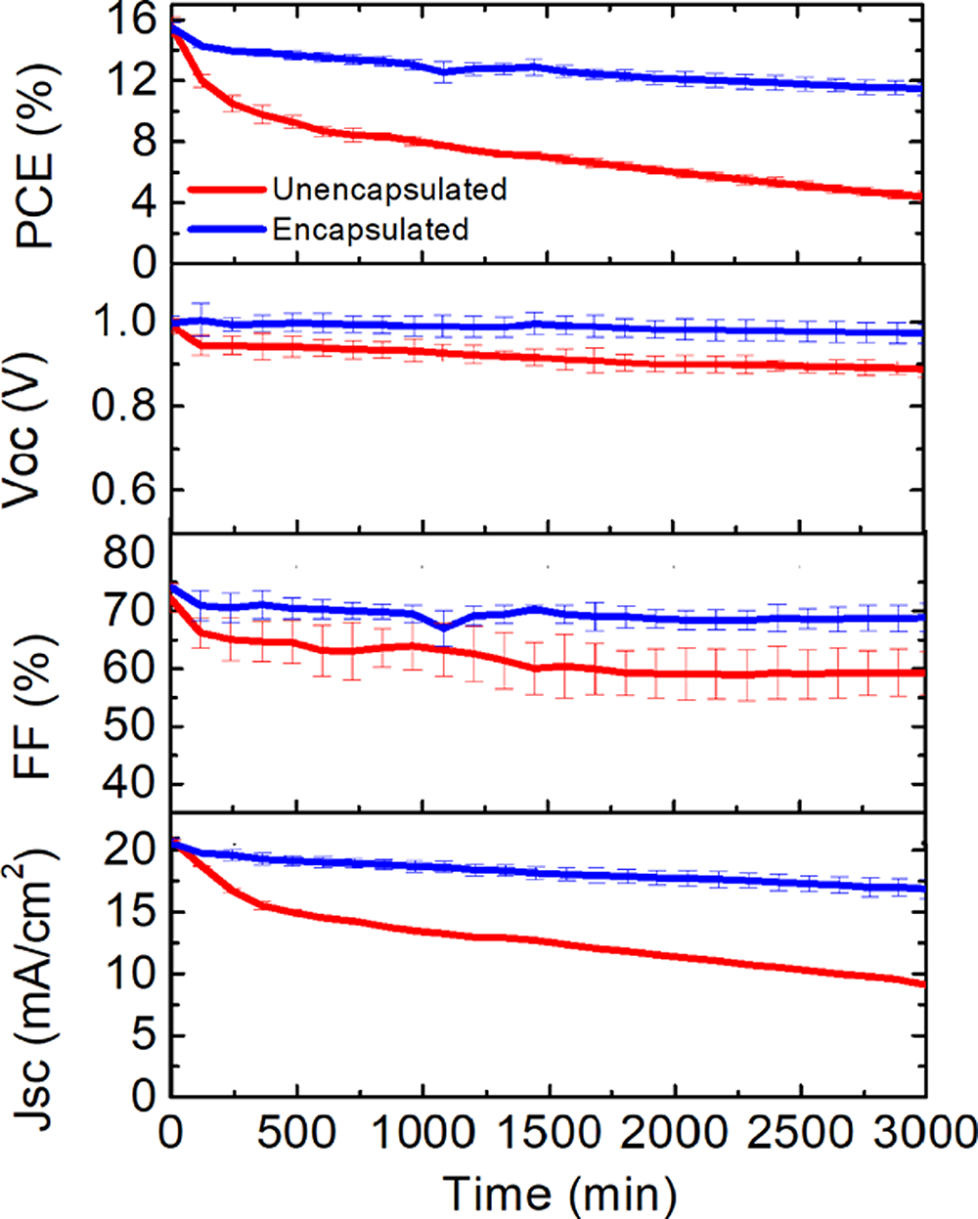
Normalized photovoltaic parameters (*V*_OC_, *J*_SC_, fill factor, and PCE in the first, second, third, and fourth graphs, respectively) extracted from light-soaking measurement of encapsulated (blue lines) and unencapsulated (red lines) devices. Value are averaged between five namely identical devices. The light-soaking stability test was accomplished under the illumination MPPT tracking system composed of a white LED array (4200 K).

To further prove the beneficial effect of PU encapsulation, we performed light-soaking (LS) measurements on both encapsulated and unencapsulated devices. During the aging test, cells are constantly operated at the MPP at room temperature under illumination; simultaneously, the photoelectrochemical performance is regularly monitored by extracting the photovoltaic parameters PCE, *J*_SC_, FF, and *V*_OC_. It is worth mentioning that the PU film is applied only onto the back of the device, whereas the illumination is provided from the front. Therefore, the radiation reaching the photoactive layer is exactly the same for both the devices being not filtered but for the front glass. Notwithstanding this, better stability of the encapsulated device is expected, with the photoinduced degradation processes being exalted by the permeation of both oxygen and water. Indeed, encapsulated devices evidenced slightly higher photostability. It should be pointed out that, likely to ISOS-D1 measurements (Figure 3c), we did not expect the “drop and recover” PCE behavior evidenced in Figure S10 (*i.e.*, aging in a laboratory environment), with the LS measurements being started after the polymerization of the PU is totally completed (i.e,. after roughly 1 week). During polymerization time, the cells were stored in controlled atmosphere to avoid any degradation: the PCE values of fresh sample were comparable to those measured at *t* = 0 in LS measurements. Figure 5 reports an average of electrical parameters of three devices normalized to the value obtained at *t* = 0 min. The significant *J*_SC_ drop recorded for unencapsulated tested PSCs strongly reduces the device performance during the endurance test. The degradation of the light-harvesting perovskite layer explains the *J*_SC_ depletion with aging. Actually, encapsulated PSCs allow one to limit the PCE drop that is observed in the stability by showing a *T*_80_ (losing 20% of the initial PCE value) equal to 250 min of the stress test, whereas the PCE of the unencapsulated cells decayed by 60% over the same period (*T*_80_ equal to 52 h). This indicates that the PU encapsulation can effectively reduce the aging effects in the PSCs and will improve the stability of PSCs in real operating conditions where light, temperature, and moisture are combined.^23^ Indeed, a possible explanation of such evidence consists of the protecting action of the polyurethane layer and its extension throughout the HTM layer (Figure 4e): this partially prevents the moisture or oxygen from diffusing throughout the HTM layer and reaching the PSK layer. Therefore, the moisture/oxygen degradation processes are partially minimized. Moreover, PU23 may fill the voids commonly present in the HTM layer, leading to more hindered diffusion of photogenerated defects.

**Figure 6:**
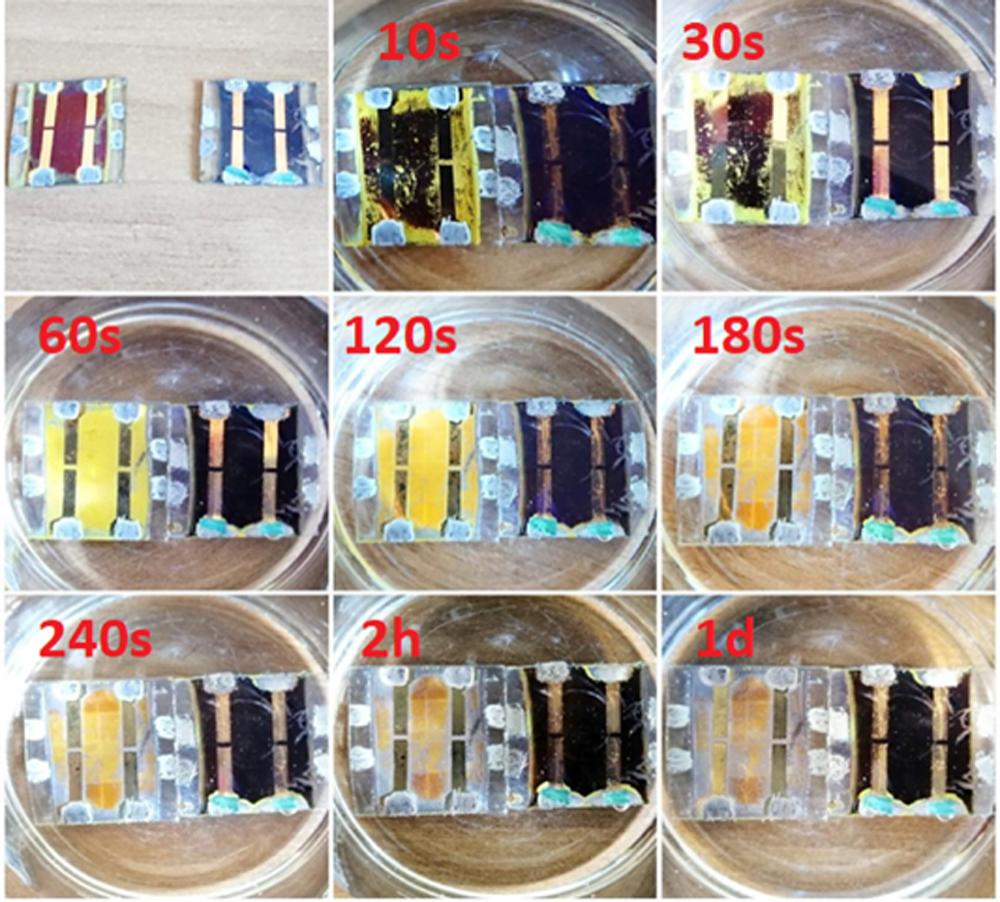
Dipping of unencapsulated (on the left) and PU23-encapsulated devices (on the right) in deionized water for different amounts of time. The first photograph refers to samples stored for 2 days (*i.e.*, polymerization time) in an ambient condition.

To further gauge the scientific and technological interest due to the implementation of PU23 as an encapsulant material in perovskite solar cells, we dipped our devices in a deionized water solution. The results were quite impressive (Figure 6): as expected, the unencapsulated device suddenly started to degrade, becoming more and more yellow; after 120 s, the entire PSK layer was converted into PbI_2_ that tended to be quite rapidly (240 s) solubilized in water; and after 5 min (approx.), the Au-based contacts were the only evidence of the original device. Remarkably, the PSK layer beneath the Au contact was degraded too. On the other hand, the encapsulated device did not suffer any degradation once immersed in water and appeared to be practically unchanged after more than 4 days. It is worth mentioning that negligible degradation of the PSK layer of encapsulated device started after 1 day (last picture in Figure 6). Yet, this degradation occurred from the silver contacts (that were not fully masked to allow the testing of the device) and, straightforwardly, it could be easily avoided by optimized engineering of the device (*e.g.*, with external contacts). Additionally, this issue would be easily avoided in module configuration. After 6 days of continuous dipping, the PSK layer of the encapsulated device was completely converted into PbI_2_ (*i.e.*, total yellowing of the device). It is important to stress how the unprotected device reached the same status after only 60 s: this means that the degradation occurred roughly 9000 times faster. This evidence opens the door to possible application of PSCs also in a water (or moisture)-rich environment, pointing out that proper encapsulation of the device is a mandatory issue to be solved toward the commercialization of this technology.

Similar results could be, in principle, also obtained with conventional “encapsulation” methods (even though there are just a few reports in the literature).^70^ Yet, as far as we are aware, *in situ*-polymerized PU-based resins are, up to date, the only technology allowing one to obtain effective encapsulation properties, being cost-effective and easily scalable, avoiding a complicated deposition method. Furthermore, being flexible, they could be effectively used also in flexible electronics (not limited to PSCs).

## 3 Conclusions

Throughout the present paper, we report, for the first time, on the direct *in situ* polymerization of polyurethane-based resins (without any additional encapsulant material) as an effective, cost-effective, and easily scalable moisture and/or oxygen barrier in perovskite solar cells (PSCs). The most promising polyurethane-based resins were selected out of six different formulations; polyurethane-based self-standing films were deeply characterized to evidence any failure due to prolonged UV or thermal (both at 100 °C and −196 °C) aging. PU23 films showed low or insignificant degradation; *i.e.*, they retain their initial morphological and optical features.

The encapsulated final devices exhibited worth-to-notice stability (over 2500 h) when stored in an ambient condition, retaining more than 90% of their initial efficiency. In the same experimental condition, the unencapsulated cell lost more than 20, 50, 80, and 99% of its initial value after 37, 125, 300, and 2500 h, respectively. The improved stability was ascribed to the extremely good moisture and oxygen barrier properties ensured from the PU film that prevented the degradation of the PSK film into PBI_2_ as proved by XRD and optical microscopy.

Very interestingly, the implementation of PU film onto the back of the device slightly enhanced the photostability of the PSCs too. The latter could be substantially improved by the employment of a protective layer onto the top of the device. Furthermore, when dipped in a deionized water solution, the encapsulated devices showed stability 9000 times higher than the unprotected counterpart. This value could be sensibly enhanced by further engineering of the electric contacts.

Overall, we proved that *in situ*-polymerized aliphatic polyurethane-based resins are a class of transparent barrier materials allowing one to obtain good retention of photoconversion efficiency over a prolonged aging period, which are of low cost, easy to synthetize, tune, and recycle, scalable, environmental-friendly, and applicable to both rigid and flexible substrates.

## 4 Experimental Section

### 4.1 Deposition of Thermosetting PUs

All the precursors are provided by SE Special Engines S.r.l. Three different diisocyanate hardeners (ICs; namely, IC1, IC2, and IC3) differing in the hexamethylene-diisocyanate (HDI)/isophorone diisocyanate (IPDI) ratios and two polyol formulations (PO; namely, PO1 and PO2) with different prepolymerization degrees were formulated (SE Special Engines S.r.l.) and employed without any further modification. UV stabilizers are dissolved into the PO formulation to enhance the stability of the resulting polymer toward high energetic radiation. The IC and PO precursors were cross-combined (in a 1:1 w/w ratio) to obtained six different polyurethane-based polymers that were characterized as self-standing films. As soon as the polymerization process is completed, the PU layer was mechanically removed from the substrate surface. The polymerization time depends on the precursor nature and quantity (Table 1). For the application as an encapsulant onto PSCs, the IC/PO (1:1 w/w ratio) mixture was directly drop-casted onto the back side of the device and then treated under continuous vacuum for 10 min to remove eventual air bubbles produced during the mixing and deposition processes. Both film thickness and uniformity are expected to influence the encapsulation properties of barrier layer. To ensure uniformity, we degassed the polyol-based precursor for 30 min under vacuum (to eliminate air bubbles and/or water traces) and then we carefully drop-casted it onto the device; on the other hand, the encapsulant thickness could be controlled by the amount of precursor mixture that was drop-casted onto the devices.

### 4.2 Characterization and Aging of PUs

Self-standings films of the more promising PU (*i.e.*, PU23) were aged in different conditions (*i.e.*, thermally stressed in an oven at 100 °C and under liquid nitrogen at −50 °C or optically stressed under continuous UV irradiation (Dymax EC-5000 Lamp)). During aging, their optical and morphological features were continuously monitored by means of both UV (UV-1700 PharmaSpec by Shimadzu) and IR (FTIR-8400 from Shimadzu) spectroscopies. The oxygen transmission rate (OTR) and water vapor transmission rate (WVTR) were measured on the self-standing polymeric films with a MultiPerm (ExtraSolution) instrument. The sample is inserted between two separated chambers and it acts as a membrane. Both the chambers are evacuated by using an inert gas (N_2_), leading to an oxygen (or water vapor) concentration lower than 1 ppm. After that, the desired gas is fluxed in the upper chamber and its concentration is measured in the bottom one until a stationary value is reached. The analyses are done, keeping both the temperature and relative humidity constant. The thermogravimetric analyses (TGA Q500 from TA Instrument) were performed on the polymeric film sealed on an aluminum plate heated from room temperature up to 800 °C with a heating rate of 20 °C/min. Before performing any DSC measurements (DSC Q200 from TA Instrument), the polymeric films were stored at 40 °C in vacuum overnight to remove residual water molecules adsorbed on the surface, and then the samples were sealed in an aluminum pan and cooled down from RT to −80 ° C. After being at that temperature for 5 min, they were heated up to 180 ° C with a heating ramp of 5 °C/min and then cooled down back to −85 ° C. This cycle was repeated twice to evidence any postcuring effect on the samples. To check the kinetic of the polymerization process, we continuously recorded the IR spectrum (Spectrum Spotlight 300 FT-IR spectrometer from PerkinElmer) of the polymerizing sample: the mixture of precursors was deposited onto an aluminum foil and the IR spectra were recorded every hour until the process was ended.

### 4.3 Preparation and Storage of PSCs

All the materials have been purchased at the highest degree of purity available. All solvent are purchased from Sigma-Aldrich if not differently specified. Perovskite solar devices were fabricated as follows: For Np-SnO_2_, the 15% tin (IV) oxide nanoparticles in H_2_O colloidal solution (Alfa Aesar) was spin-coated in air onto glass/ITO substrates (Kintex 15 Ω/cm^2^) at 6000 rpm for 45 s and then annealed in air at 100 °C for 1 h. To make the perovskite precursor solution, 547.4 mg mL^–1^ PbI_2_ (TCI), 87.1 mg mL^–1^ PbBr_2_ (TCI), 21.6 mg mL^–1^ MABr (GreatCell Solar), 166 mg mL^–1^ FAI (GreatCell Solar), and 19.4 mg mL^–1^ CsI (Sigma-Aldrich) were dissolved in mixed *N*,*N*-dimethyl sulfoxide (DMSO) and *N*,*N*-diethyl formamide (DMF) solvents (1:3.16 by volume) by stirring for 24 h at room temperature. The as-prepared precursor solution was then spin-coated in a glovebox (GB) onto the tin oxide film with two-step spinning: first 1000 rpm for 10 s and then 5000 rpm for 30 s; 7 s before the end of the second spin coating step, 0.2 mL of chlorobenzene was dropped on the substrates. Afterward, the perovskite layer (about 600 nm) was treated at 100 °C for 50 min in GB. The hole-transporting material (HTM), a solution of spiro-OMeTAD (from Borun) in chlorobenzene (CB) (73.5 mg/mL) with additives of lithium bis-(trifluoromethyl sulfonyl)imide (Sigma-Aldrich) in acetonitrile (16.6 μL, 520 mg/mL), cobalt additive (7.2 μL, FK209 from Sigma-Aldrich, 0.25 M in acetonitrile), and 4-*tert*-butylpyridine (27 μL, from Sigma Aldrich), was spin-coated at 2000 rpm for 20 s in air. Finally, the cells were completed by thermal evaporation of Au (80 nm) as the top electrode. Both PU-encapsulated and unencapsulated devices were stored in a laboratory shelf (*i.e.*, indoor illumination; temperature range: 18–30 °C; RH ranging from 28 to 65%) without any additional protection and tested every 3–4 days. It is worth mentioning that a univocal testing protocol for PSCs has been only recently established.^62^ Before that, scientists usually refer to protocols commonly adopted for OPVs.

### 4.4 Characterization of (Un)encapsulated Devices

An X-ray diffraction apparatus and optical microscope (Leica DM2500; maximum magnification, 200×) were employed to evaluate the degradation of (un)encapsulated devices during shelf life. The powder X-ray diffraction patterns (VTXRD) were collected with an X’Pert PRO MPD diffractometer from PANalytical working in Bragg–Brentano geometry equipped with a Cu K_α_ source. Scattered photons were revealed by an X’celerator linear detector equipped with a Ni filter to attenuate K_β_. WAXS experiments were carried out on a Bruker D8 Advance with a DaVinci design diffractometer (angle-dispersive). The diffractometer is equipped with a Mo K_α_ X-ray tube (λ = 0.7107 Å). The scattered intensity was gathered with the Lynxeye XE Energy-Dispersive 1-D detector. For the electrical characterization of the devices, we employed a custom-made system, which allows one to simultaneously measure all the pixels of a device plate. They were carried out under a class A solar simulator (ABET Sun 2000) at a flux density of 1000 W m^–2^ (scan rate of 60 mV/s in reverse mode) with artificial solar spectrum AM 1.5G whose lamp was calibrated with a pyranometer. The devices were masked with an aperture of 0.1 cm^2^ to define the active working area. All electrical contacts are covered with silver paste. The external quantum efficiency (EQE) spectra were collected as a function of wavelength using an optical power density-based measurement system (Arkeo from Cicci Research s.r.l.) under the irradiation of 300 W xenon lamp (Mod.70612, Newport). The light-soaking stability test was accomplished under the illumination MPPT tracking system (Cicci Research s.r.l.) composed of a white LED array (4200 K) tunable up to an optical power density of 2000 W m^–2^ and a high-speed source meter unit.

## Supporting Information Available

Polymer-based encapsulant for PSCs (Table S1); thermogravimetric analyses of thermosetting PU-based resins (Figures S1–S3); differential scanning calorimetry (Figures S4–S6); scheme of the deposition process (Figure S7); photograph of devices (Figure S8); photoelectrochemical parameters of unencapsulated devices (Figure S9); photoconversion efficiency as a function of aging and polymerization time (Figure S10) (PDF)
