# Supplementary material for: Thermosetting Polyurethane Resins as Low-Cost, Easily Scalable, and Effective Oxygen and Moisture Barriers for Perovskite Solar Cells
Source: ACS Appl Mater Interfaces. 2020 Nov 25;12(49):54862–75. doi: 10.1021/acsami.0c17652 (PMC8016164; doi:10.1021/acsami.0c17652)
Supplement: Supplementary file 1 — am0c17652_si_001.pdf [file am0c17652_si_001.pdf]

Supporting Information

Thermosetting polyurethanes resins as low-cost, easily scalable and effective oxygen and moisture barriers for Perovskite Solar Cells

Matteo Bonomo, Babak Taheri, Luca Bonandini, Sergio Castro-Hermosa, Thomas M. Brown, Marco Zanetti, Alberto Menozzi, Claudia Barolo*, and Francesca Brunetti*

Dr. M. Bonomo, Prof. M. Zanetti and Prof. C. Barolo
Department of Chemistry and NIS Interdepartmental Centre, University of Turin, Via Pietro Giuria 7, 10125 Turin, Italy

Dr. B. Taheri, Dr. S. Castro-Hermosa, Prof. T.M. Brown and Prof. F. Brunetti
CHOSE (Centre for Hybrid and Organic Solar Energy), Department of Electronic Engineering, University of Rome Tor Vergata, Via del Politecnico 1, 00133 Rome, Italy

L. Bonandini and A. Menozzi
S.E. Special Engines S.r.l., Strada del Cascinotto, 163, 10156 Torino, Italy

Prof. M. Zanetti and Prof. C. Barolo
ICxT Interdepartmental Centre, Università degli Studi di Torino, Lungo Dora Siena 100, 10153 Torino, Italy

Corresponding authors' email: claudia.barolo@unito.it and francesca.brunetti@uniroma2.it

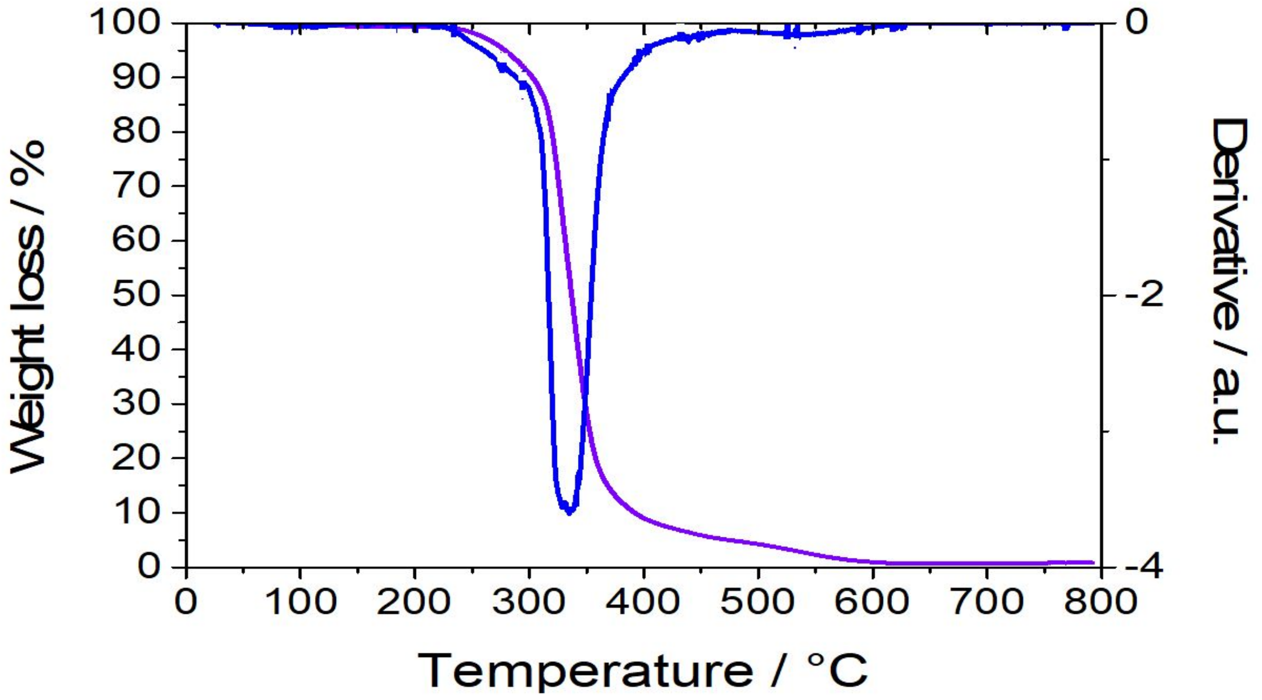

Figure S1. Thermogravimetric analyses (purple) and differential thermogravimetric analyses (blue) of the self-standing film of PU21 polymer. Scan rate 5 °C/min.

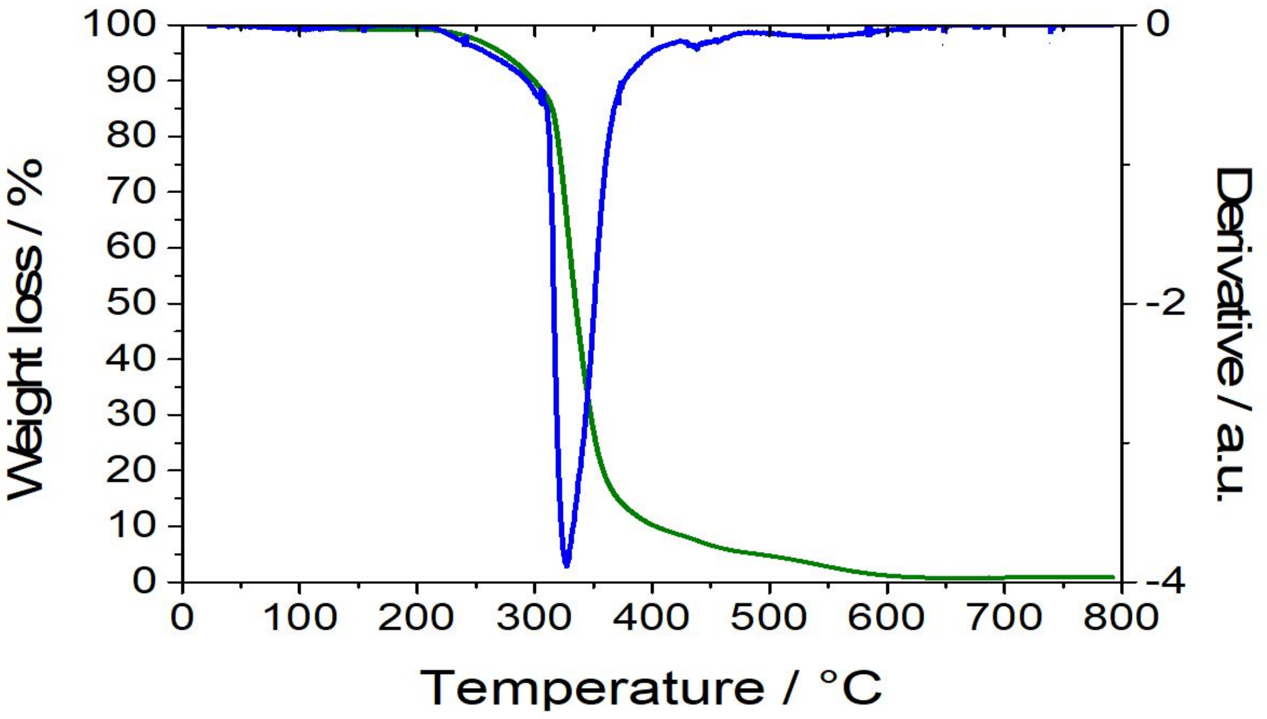

Figure S2. Thermogravimetric analyses (red) and differential thermogravimetric analyses (blue) of the self-standing film of PU22 polymer. Scan rate 5 °C/min

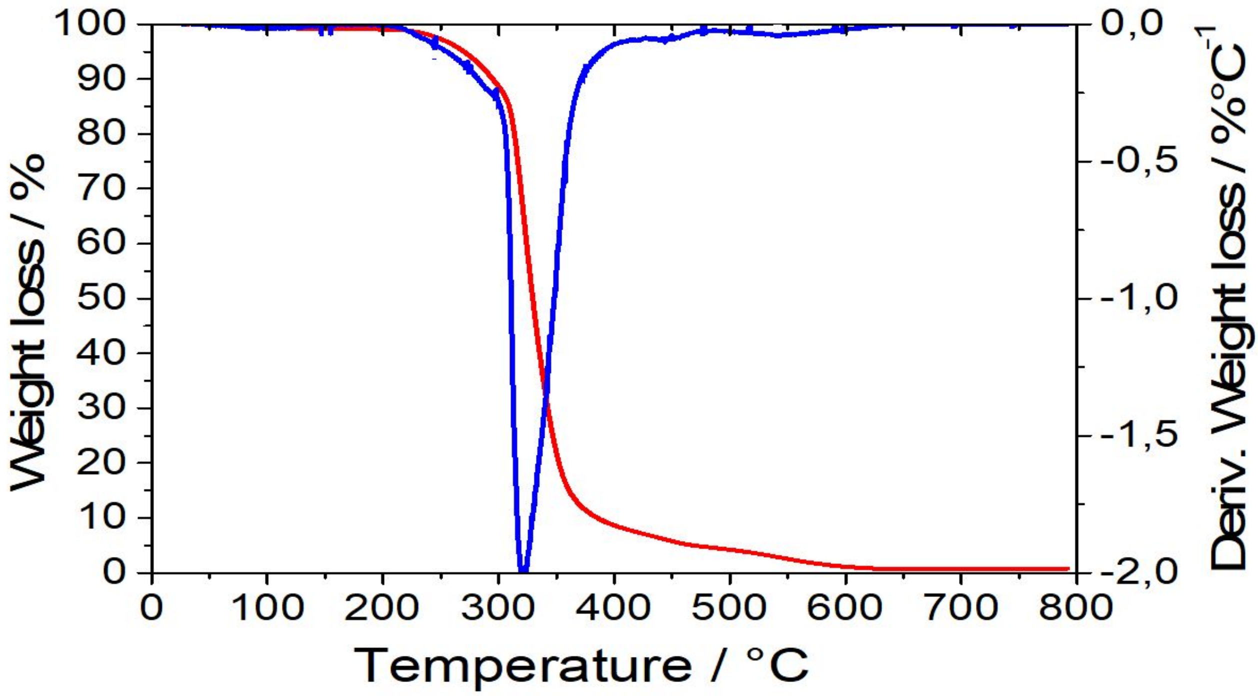

Figure S3. Thermogravimetric analyses (red) and differential thermogravimetric analyses (blue) of the self-standing film of PU23 polymer. Scan rate 5 °C/min

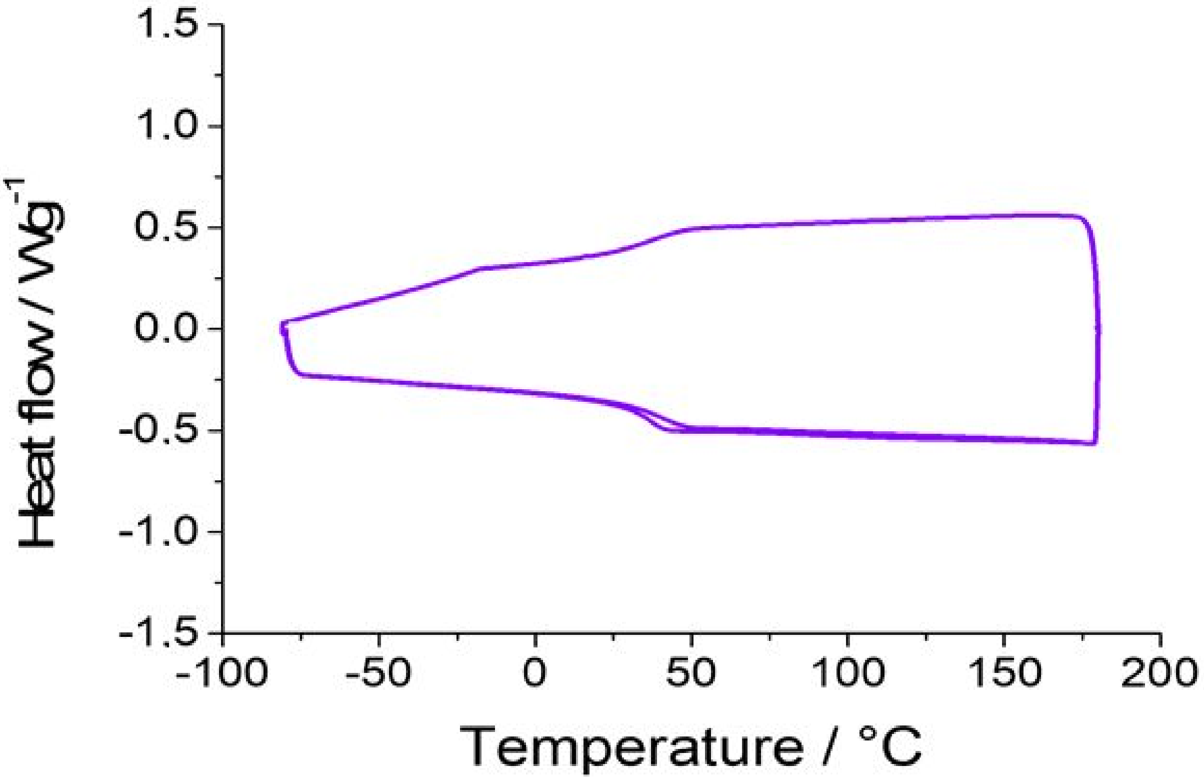

Figure S4. Differential Scanning Calorimetry (DSC) analyses of the self-standing film of PU21. There is not any evidence of post-curing process. Scan rate 5 °C/min

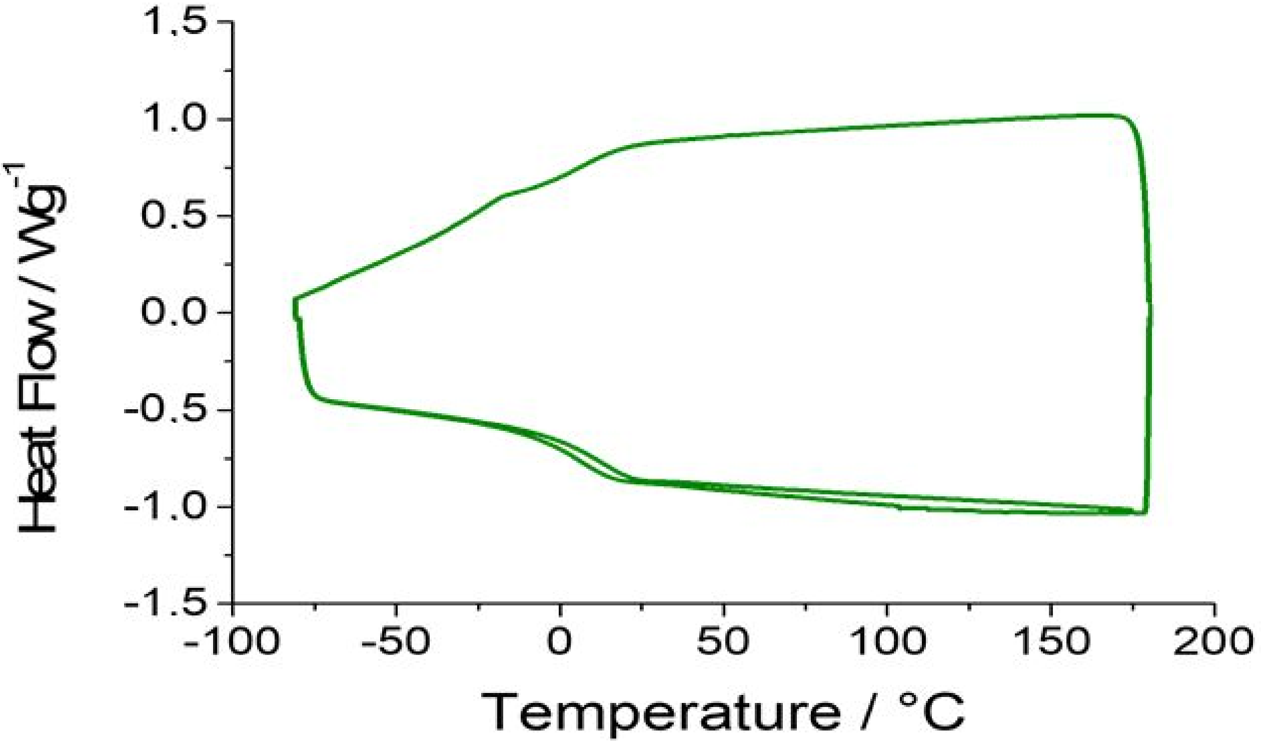

Figure S5. Differential Scanning Calorimetry (DSC) analyses of the self-standing film of PU22. There is not any evidence of post-curing process. Scan rate 5 °C/min.

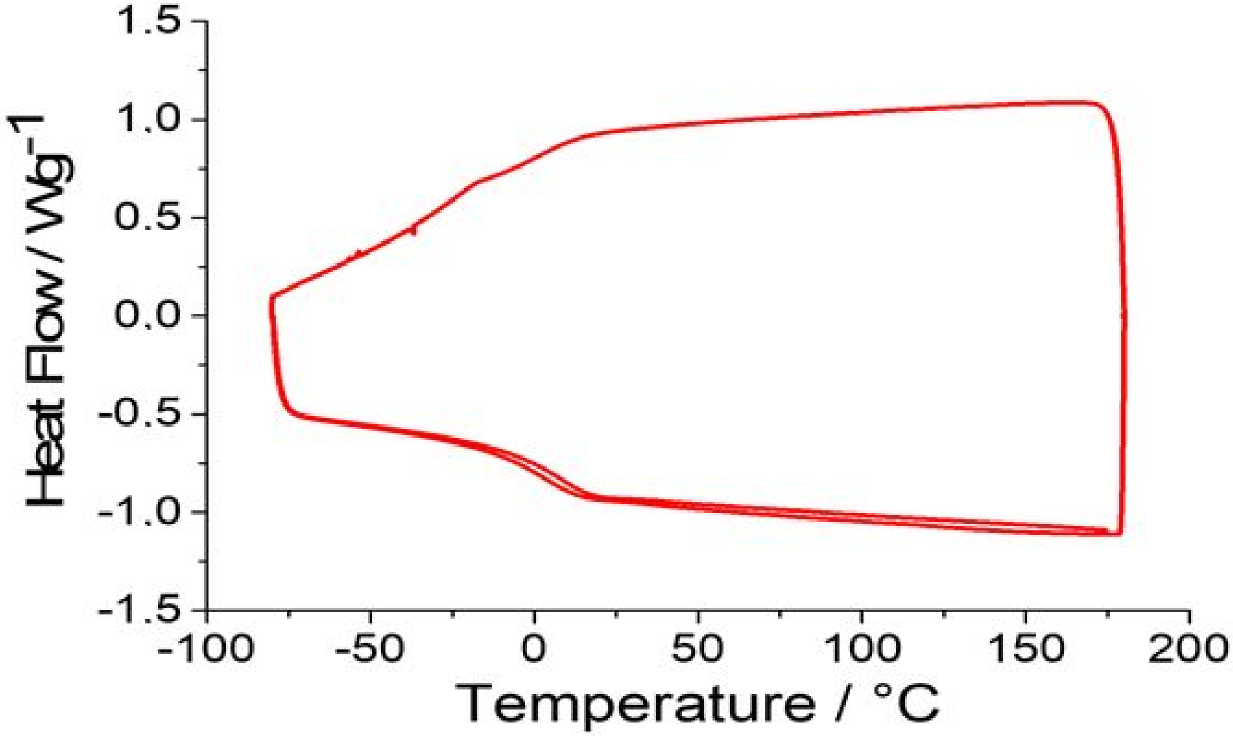

Figure S6. Differential Scanning Calorimetry (DSC) analyses of the self-standing film of PU23. There is not any evidence of post-curing process. Scan rate 5 °C/min.

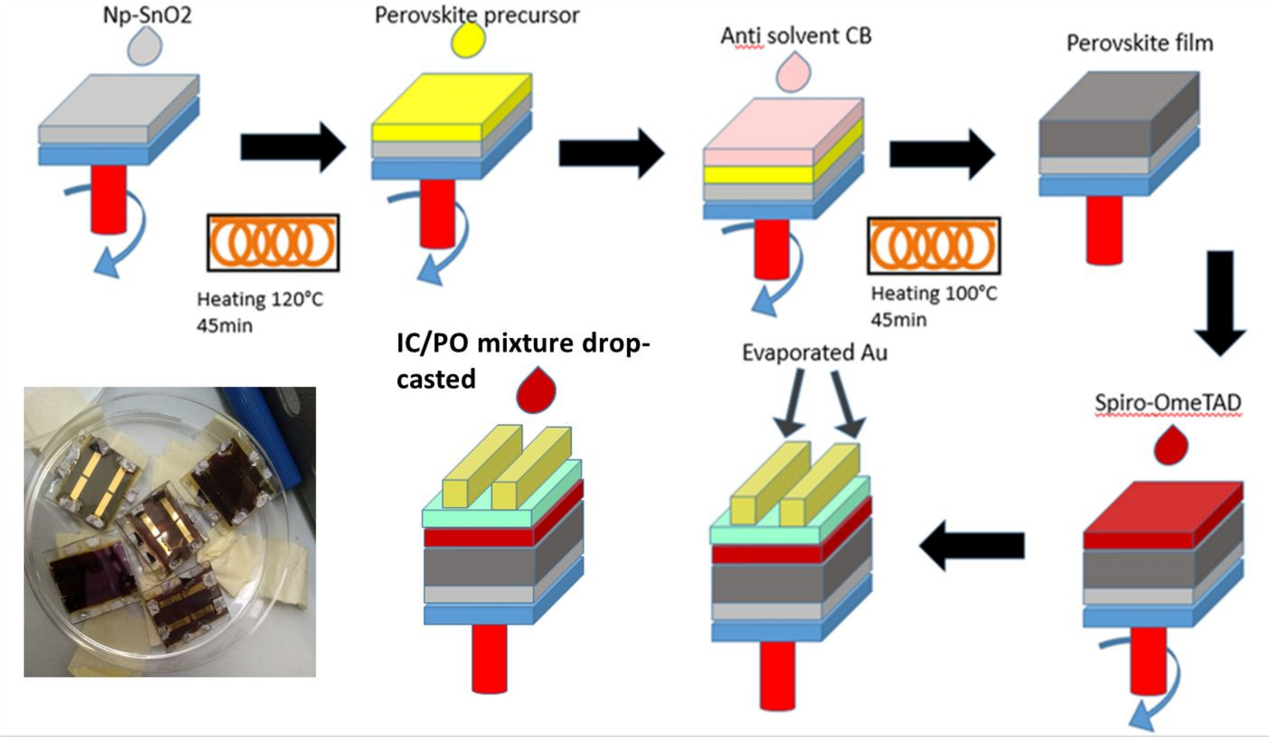

Figure S7. Scheme representing the deposition process to obtain a n-i-p Perovskite Solar Cells.

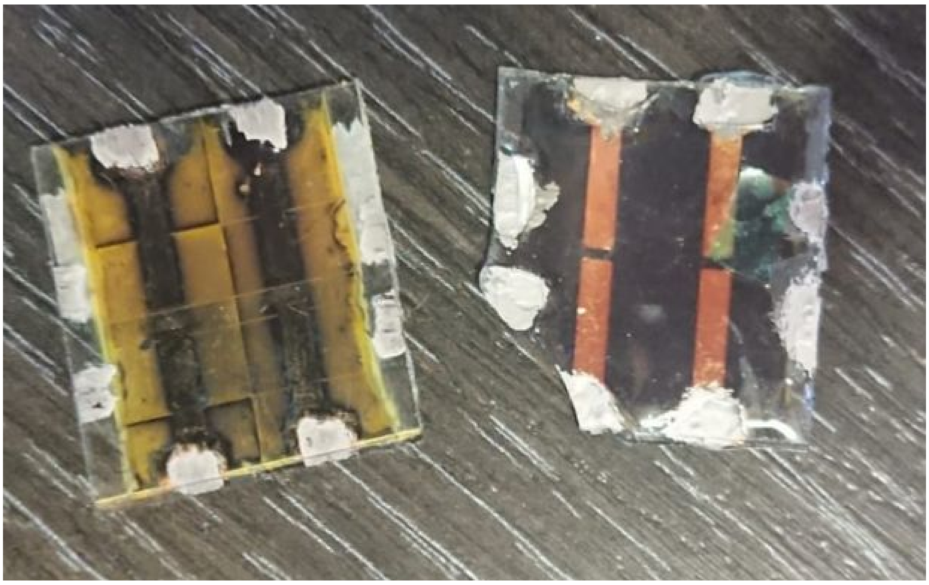

Figure S8. Digital photograph of unencapsulated (on the left) and encapsulated (on the right) after two days of storing in controlled atmosphere. The encapsulated device remains stable up to 100 dyas.

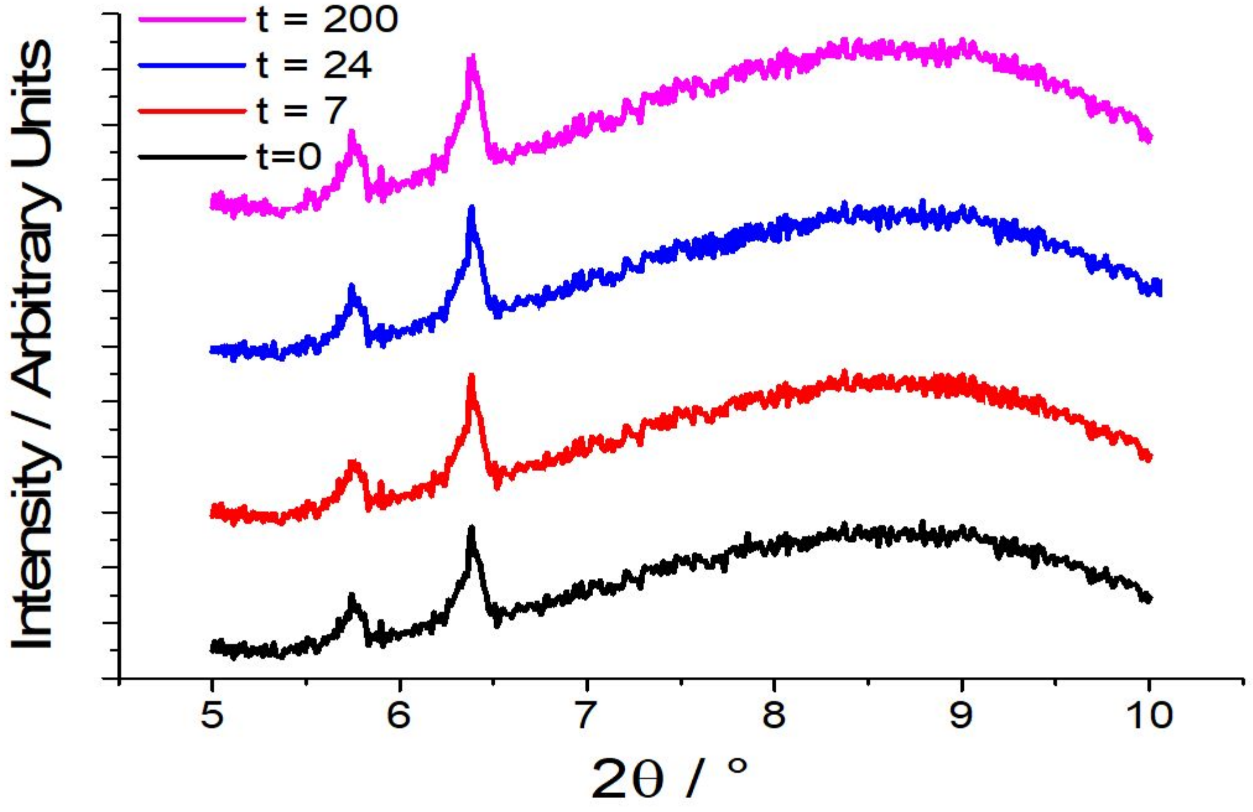

Figure S9. Diffractograms of encapsulated samples after 1h (black trace), 7h (red trace), 24h (blue trace) and 200h (purple trace) of ageing time (stored at RH < 60% and T ranging from 20 to 30 °C);

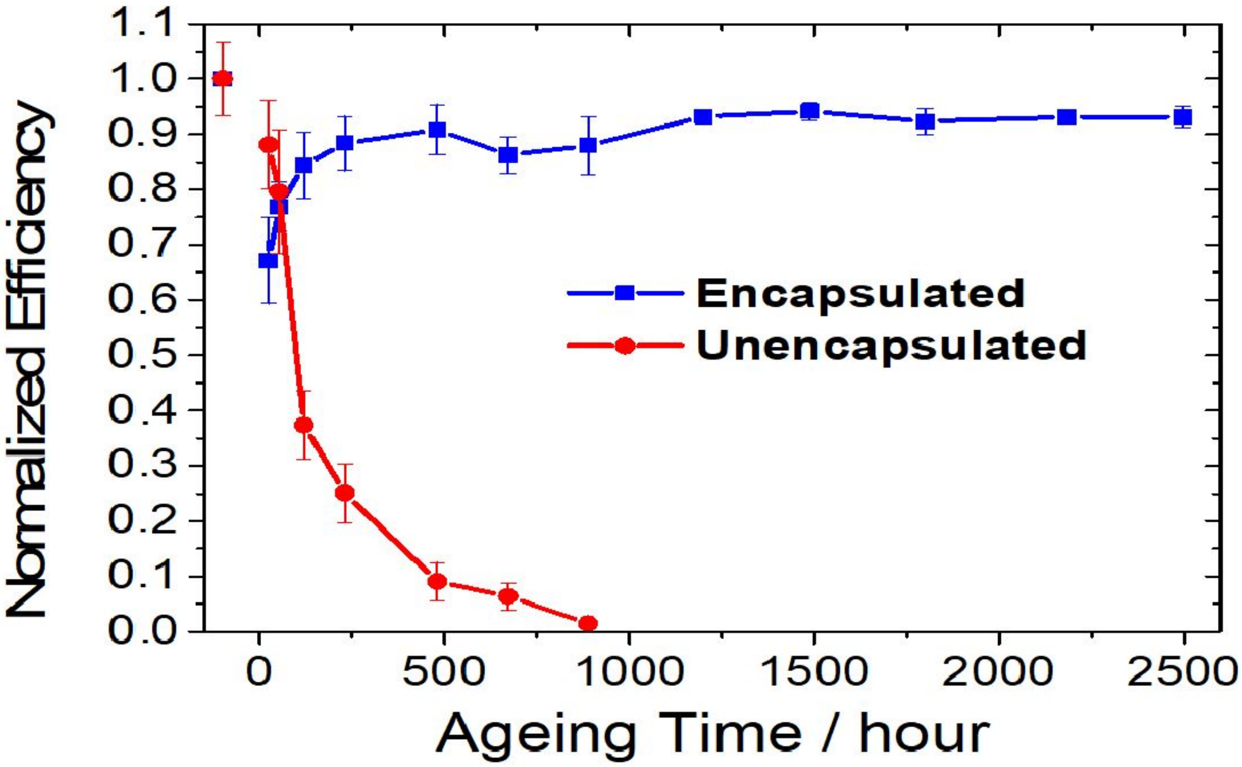

Figure S10. Comparison between normalized power conversion efficiency (measured under 1 Sun AM1.5G) vs ageing time of unencapsulated (red dots) and PU23-encapsulated (blue squares) devices; ageing was performed stored under ambient light (400-1000 lux) controlled humidity (28-65%) and temperature (18-30 °C).

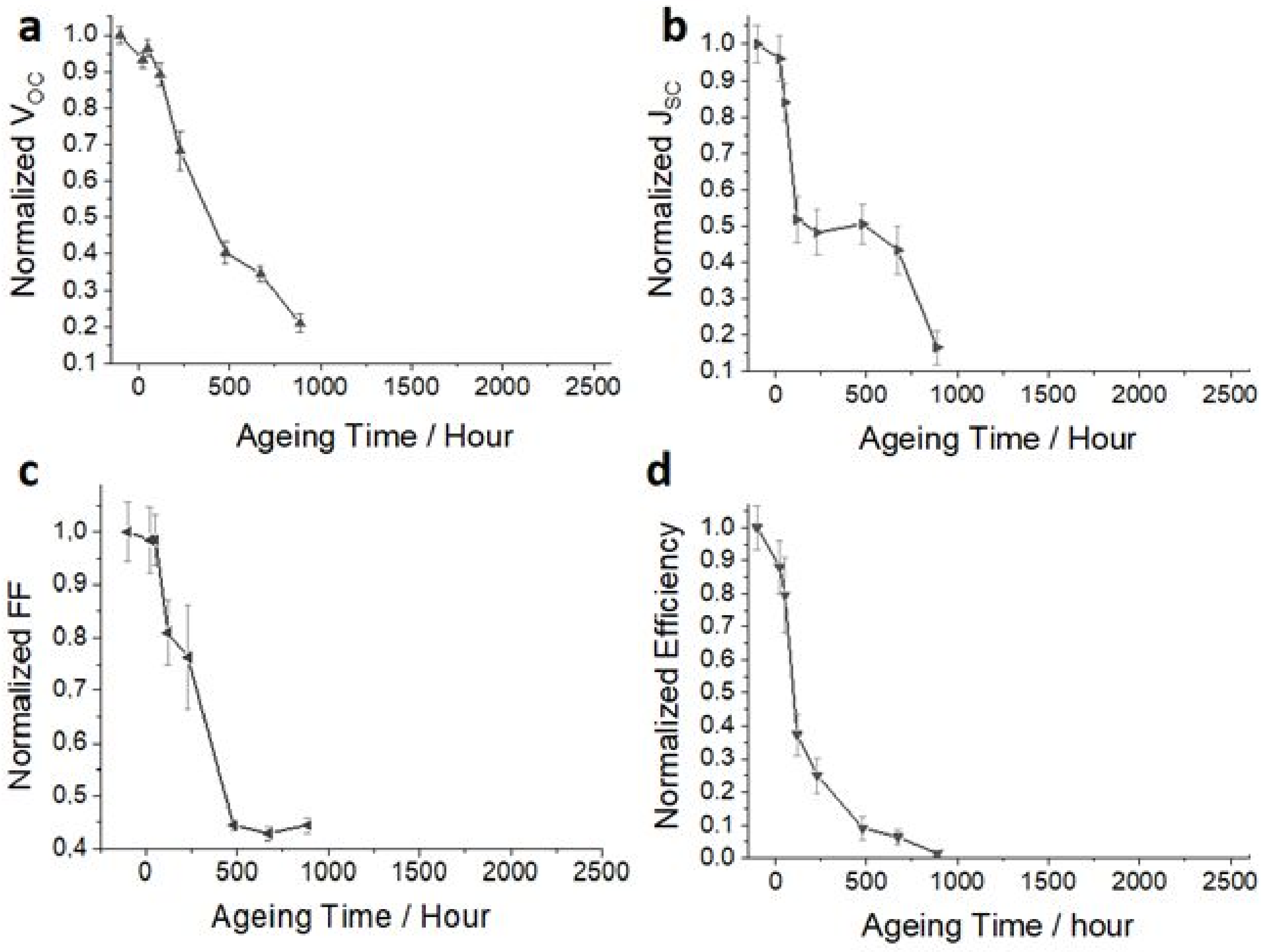

Figure S11. Normalized photoelectrochemical parameters (i.e. V_{OC} (a); J_{SC} (b) and PCE (c)) of unencapsulated devices within the ageing period (measured under 1 Sun AM1.5G).

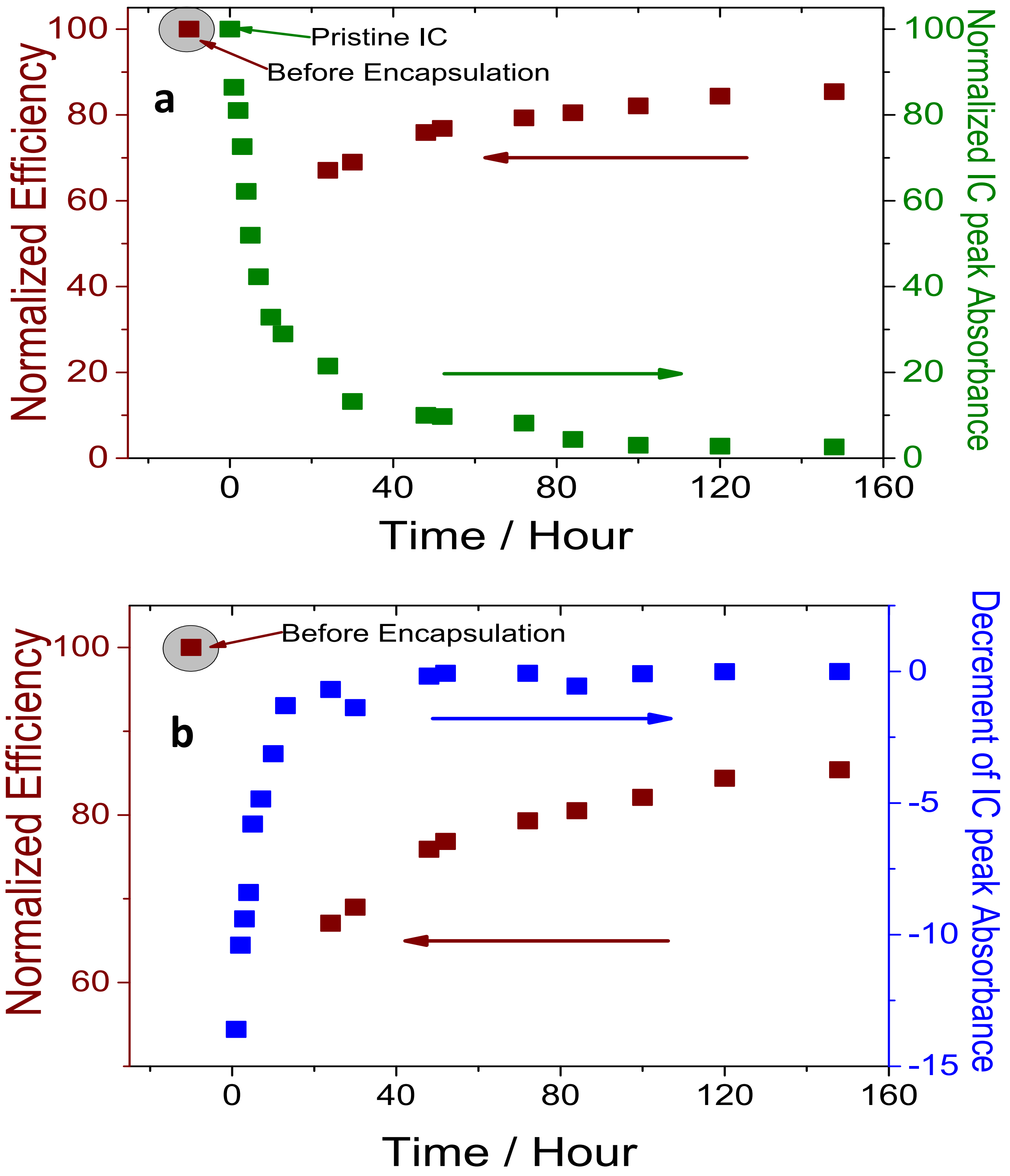

Figure S12. Comparison between the trend of the photoconversion efficiency (wine squares) and the intensity of the isocyanate peak in ATR spectra (green squares) as a function of (ageing and polymerization, respectively) time (a). In (b) the photoconversion efficiency (wine squares) is compared with percentage decrement of the intensity of isocyanate peak (blue squares).

As reported in the main text, the trend of PCE with the ageing time is not straightforward. An initial sharp drop in efficiency (in the first 24 hours) is followed by a slow but constant recover of the efficiency, reaching a constant value after 480 hours of ageing. In figure 4d is reported the evolution of ATR spectra of PU precursor mixture during time. A t=0, *i.e.* when the IC and PO precursors are mixed, the peak located at 2250 cm^{-1} ascribed to the stretching of isocyanate moiety (I_{IC}) is clearly present. Then, the peak starts to decrease following on the reaction between the latter and the hydroxyl moieties. The decrease of this peak is coupled with the increase of the peak at 1750 cm^{-1}, characteristic of the stretching on NH group of the polyurethane structure. At first, polymerization reaction occurs quite fast and I_{IC} decrease at 30% and 20% of its initial value after just 13 and 24 hours, respectively. It is worth to mentioning that, within the first day, it is not possible to measure the photoconversion efficiency of encapsulated device being the first step of the polymerization not complete and the cell not wieldy. After 24 hours, we recorded the efficiency of the device and it was 67% of the initial value. In the following 48 hours (two days) the device slightly but constantly recovers its efficiency reaching roughly 80% of starting PCE. As evidenced in figure S10a, the recovery trend is associated to a sizeable slowdown of the polymerization kinetic (*i.e.* I_{IC} = 8%). The latter further slackens in the following days (I_{IC} =3% after 100 hours) and it could be considered complete only after 480 hours. As far as I_{IC} decrease, the photoconversion efficiency of continues to increase reaching 85% and 91% of its initial value after 148 and 480, respectively. The highlighted trend is a crucial evidence on the effect of polymerization time onto the drop and the successive recover in the PCE of encapsulated device. This trend is even clearer if compared to the differential decrease of I_{IC}, *i.e.* the difference between the intensity value of two successive point, figure S10b.

We are aware that further (and possibly *in situ* and *in operando*) analyses are required to strongly prove our hypothesis (*i.e.* the dependence of PCE trend with respect to the kinetic of PU polymerization) but the latter is strongly consistent with experimental data.

Table S1. Most performing polymeric material employed as encapsulant in Perovskite Solar Cells.

Encapsulant	Deposition Method and Ambient	Film Propertie s	Test	Reference	Notes
PIB + Glass	GB		85/85 > 540 h	10.1021/acsami.7b0762 5	
PVP + Epoxy + Glass	SC<br>GB		1Sun<br>T80 = 750 h	10.1002/aenm.2018012 34	
Thermoplasti c PUs + Glass	Deposition of hot-melt films		Outdoor<br>> 2100 h	10.1002/adfm.2018091 29	Cured at 80° C
Epoxy + Multilayer Barrier	Lam.<br>GB		Shelf life<br>25 °C/35%<br>T80=840h	10.1002/aelm.20180097 8	Self-curing (RT)
UV-curable adhesive with Paraffin	Glass cover with paraffin<br>Ambient	WVTR 4<br>CA 107.4	1000h under MPPT	10.1002/aenm.2019024 72	UV-curing
Adamantate	MW plasma reactor<br>Ambient		Pured in H_2O<br>< 120 s<br>RH 85%<br>T80 < 24 h	10.1021/acsami.7b1782 4	
PMMA	SC in solvent<br>GB	WVTR 55<br>CA 77	100 °C (Abs % monitoring)<br>> 200 h	10.1039/C7RA06002E	No Photovoltai c Analyses
PC	SC in solvent<br>GB	WVTR 115<br>CA 73.5	100 °C (Abs % monitoring)<br>> 100 h	10.1039/C7RA06002E	No Photovoltai c Analyses
poly(p-chloro-xylylene) (Parylene-C)	vaporization, pyrolysis and polymerizatio n at 20 ◦C	WVTR n.a.<br>CA 121	25 °C/RH 50%.<br>T80 > 196h	10.1038/s41598-019-51945-9	

PMP	SC in solvent GB	WVTR 775 CA 106	100 °C (Abs % monitoring) > 50 h	10.1039/C7RA06002E	No Photovoltaic Analyses
V570-doped fluoropolymer	SC in solvent GB	n.a. CA 111	Outdoor ageing > 175 days	10.1126/science.aah4046	UV-curing
Thermosetting PUs	DC (no solvent) Ambient	WVTR 66 CA 102	Lab environment (1000 lux) > 2500 h	This Work	Self-curing (RT)

Lam = lamination; SC = Spin coating; DC = Drop casting; GB = Glove Box; MW = Microwave; CA = Contact Angle; WVTR = Water Vapor Transmission Rate; RT = Room Temperature; PIB = polyisobutylene; PU = polyurethane PVP = Polyvinylpyrrolidone;; PMMA = Poly(methyl methacrylate); PC = polycarbonate; PMP = Polymethylpentene; WVTR and CA values are reported in g m^{-2} day^{-1} for fully polymeric encapsulation only.
